# Over 65% Sunlight Absorption in a 1 μm Si Slab with Hyperuniform Texture

**DOI:** 10.1021/acsphotonics.1c01668

**Published:** 2022-03-22

**Authors:** Nasim Tavakoli, Richard Spalding, Alexander Lambertz, Pepijn Koppejan, Georgios Gkantzounis, Chenglong Wan, Ruslan Röhrich, Evgenia Kontoleta, A. Femius Koenderink, Riccardo Sapienza, Marian Florescu, Esther Alarcon-Llado

**Affiliations:** † Center for Nanophotonics, AMOLF, Science Park 104, 1098XG Amsterdam, The Netherlands; ‡ Department of Physics, Advanced Technology Institute, University of Surrey, GU2 7XH Guildford, United Kingdom; § Advanced Research Center for Nanolithography, Science Park 106, 1098XG Amsterdam, The Netherlands; ∥ The Blackett Laboratory, Department of Physics, Imperial College London, London SW7 2BW, United Kingdom

**Keywords:** ultrathin photovoltaics, light trapping, hyperuniform correlated disorder

## Abstract

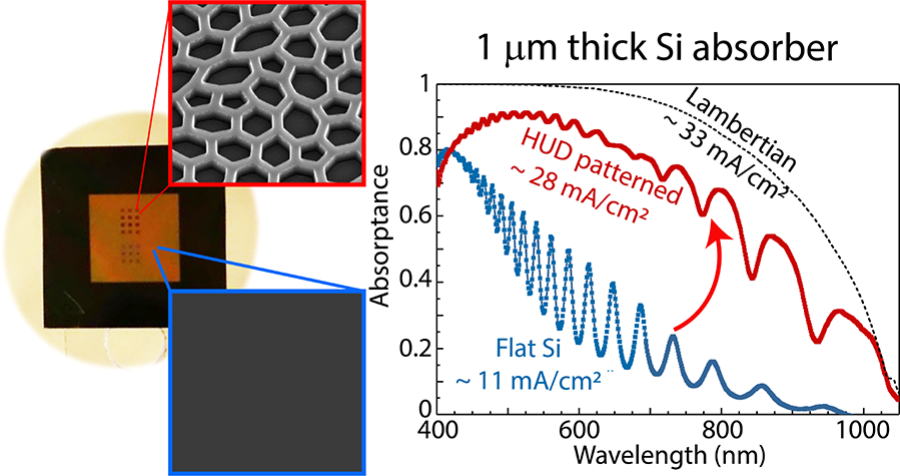

Thin, flexible, and invisible solar cells will be a ubiquitous technology in the near future. Ultrathin crystalline silicon (c-Si) cells capitalize on the success of bulk silicon cells while being lightweight and mechanically flexible, but suffer from poor absorption and efficiency. Here we present a new family of surface texturing, based on correlated disordered hyperuniform patterns, capable of efficiently coupling the incident spectrum into the silicon slab optical modes. We experimentally demonstrate 66.5% solar light absorption in free-standing 1 μm c-Si layers by hyperuniform nanostructuring for the spectral range of 400 to 1050 nm. The absorption equivalent photocurrent derived from our measurements is 26.3 mA/cm^2^, which is far above the highest found in literature for Si of similar thickness. Considering state-of-the-art Si PV technologies, we estimate that the enhanced light trapping can result in a cell efficiency above 15%. The light absorption can potentially be increased up to 33.8 mA/cm^2^ by incorporating a back-reflector and improved antireflection, for which we estimate a photovoltaic efficiency above 21% for 1 μm thick Si cells.

Micrometer-thick silicon photovoltaics (PV) promises to be the ultimate cost-effective, reliable, and environmentally friendly solution to harness solar power in urban areas and space, as it combines the low cost and maturity of crystalline silicon (c-Si) manufacturing^1^ with the low weight and mechanical flexibility of thin films.^2−4^ Efficient light trapping in ultrathin c-Si is of utmost importance when the film is thinner than the absorption length. Indeed, due to the indirect bandgap of c-Si, inefficient absorption currently hampers the thinning of Si cells below ∼100 μm, which is crucial to enable flexible, lightweight, and lower cost c-Si PV.^1,5,6^ 3D nanophotonic architectures are necessary for reducing the cell thickness as conventional antireflection coatings and multilayers can only prevent light reflection via impedance matching of the solar cell and air, but do not extend the light paths in the Si cell that are required for efficient photon absorption.^7,8^

Naively, one might think that 3D patterns like random rough surfaces (Asahi pattern) or inverted pyramids as applied in standard thick Si solar cells also will be effective for thin silicon. Indeed, very recently light-trapping performance at and even beyond the Lambertian scattering limit has been reported^9−12^ for exquisitely optimized periodic arrays of inverted pyramids and paraboloids in ca. 10 μm thick membranes. However, intrinsically the feature size for this fully 3D design approach has to be larger than the longest wavelength that is to be trapped, leading to indentations larger than 1.1 μm both laterally and in depth into the silicon. Therefore, this approach is impractical for Si thicknesses significantly smaller than the longest scattering wavelength (thickness <1.1 μm). This imposes the need for new paradigms in nanophotonic light trapping with nanotextures applicable to wavelength-thick cells.

There is still no unanimously agreed best strategy for the designing of light trapping nanotextures for PV. On one hand periodic patterning to elicit grating diffraction into a thin PV slab, whether by adding plasmonic^13−17^ or dielectric^18−20^ structures, can reduce reflection and simultaneously scatter light in the plane of the thin film. However, this strategy generally only works at discrete wavelengths and specific angle of incidence, due to the discrete crystal momenta of gratings and étendue conservation.

Conversely, fully random patterning can scatter the light over a broad angular range and over a large range of wavelengths due to the large rotational and translational symmetry, but the effectiveness is limited by the fact that the spatial frequencies in the random pattern do not specifically favor scattering into the thin film architecture.^21^ For this reason, it has been proposed to exploit correlated disordered media, which have indeed been shown to outperform both random roughening and periodic patterning for light trapping.^22−31^ Although many designs have been presented so far, it is still an open question how to reach an optimal design. For instance, it is not settled if the best design is obtained when starting the optimization from a periodic or from a random structure.

Hyperuniformity has recently emerged as a new framework to engineer light scattering and diffraction in a rational manner. Hyperuniform disordered (HUD) media are statistically isotropic and possess a constrained randomness such that density fluctuations on large scales behave more like those of ordered solids, rather than those of conventional amorphous materials.^32−37^ HUD patterns naturally arise in many physical systems, from the mass distribution in the early universe,^38^ structure of prime numbers,^39^ hydrodynamics,^40^ structure of amorphous ices,^41^ sheared sedimenting suspensions,^42^ to wave localization^43^ or colloidal packing.^44^ When translated into photonic materials, HUDs exhibit large and robust photonic band gaps as in photonic crystals, but are both complete and isotropic.^35^ As a result, HUDs display allowed modes that can propagate through the structure in an isotropic fashion as in random media. HUDs are a highly flexible platform to control light transport, emission, and absorption in unique ways, beyond the constraints imposed by conventional photonic architectures,^37,45−49^ for the design of freeform waveguides,^50^ high-quality factor resonant defects and arbitrarily high-order power splitters,^51,52^ hollow-core fibers,^53^ and photonic bandgap polarizers^54^ among others.

In this work we experimentally demonstrate that light absorption in a 1 μm-thick silicon slab is enhanced more than 2-fold in the wavelength range from 400 to 1050 nm when textured with optimized HUD-based patterns compared to the unpatterned slab. The resulting absorption is the highest demonstrated so far in a Si slab as thin as 1 μm. This record value is achieved by *k*-space engineering of HUD patterns with a tailored scattering spectrum and diffractive coupling of solar irradiation into guided modes of the Si slab. Using our strategy to light management, we investigate PV efficiency by focusing on the trade-off between light trapping and increased carrier recombination given by the nanotextures. We find that the effect of increased surface-induced charge carrier recombination on the open circuit potential can be fully compensated by the large photocurrents. A detailed PV efficiency estimation reveals that efficiencies above 20% can be obtained for several optimized HUD designs and state-of-the-art Si PV technologies. This is a highly remarkable efficiency for such a thin indirect-bandgap material, which together with the fact that lower grade raw Si material can be used in such thin devices, establishes a new breakthrough in thin lightweight and flexible solar cells.

## Results

### Light Absorption in Films with Disordered Hyperuniform Patterns

**Figure 1:**
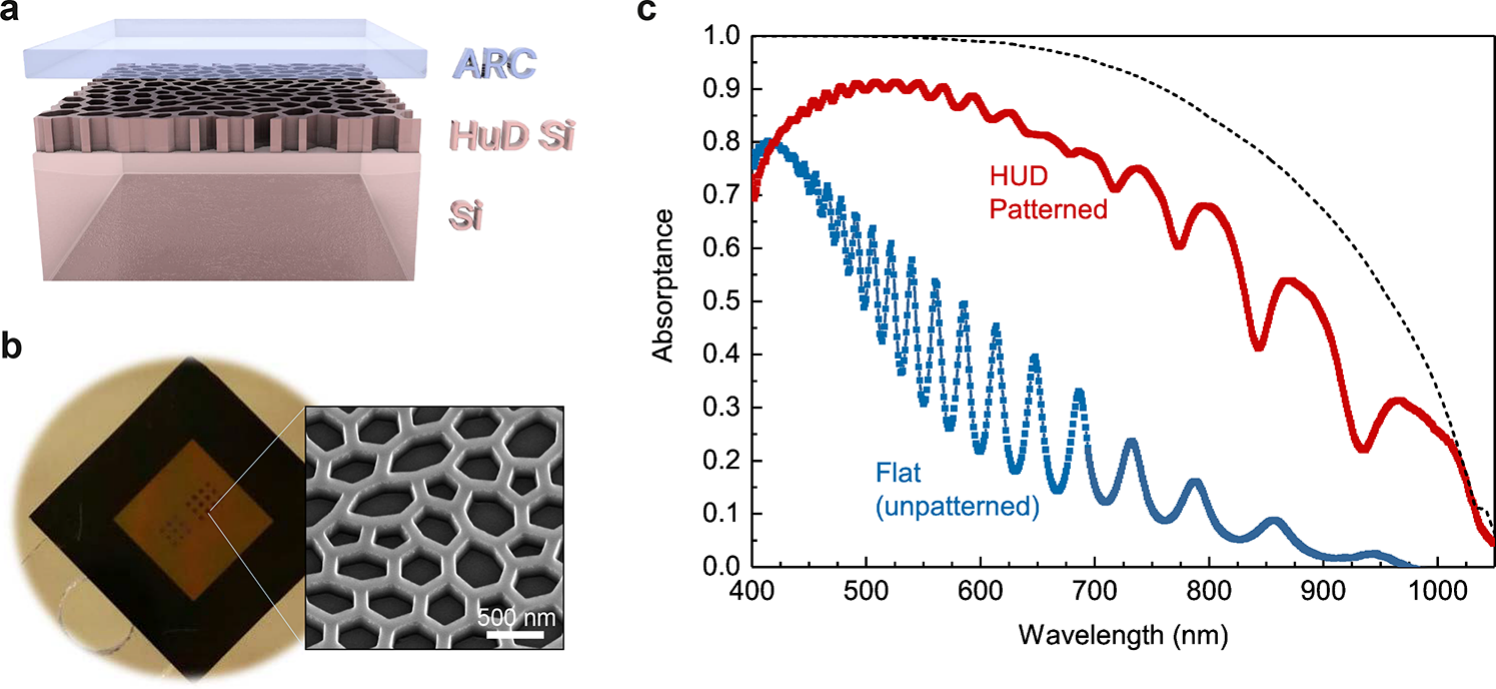
Ultrathin light absorber design. (a) Schematic representation of the ultrathin light absorber consisting of a 1 μm-thick silicon film with the HUD pattern on the top surface (∼200 nm thick) to improve light trapping. The pattern is infiltrated with a lower refractive index material (*n*_LRM_), which is also used in a top flat layer, ARC, to reduce reflectance (50–100 nm in thickness). Note that the ARC layer is depicted floating above the nanopattern in the image only for clarity purposes. (b) Optical image of the silicon membrane sample supported by a thick silicon frame (1 × 1 cm^2^), where the textures have been fabricated. Inset: Scanning electron image of the as-fabricated Si membrane with the optimized honeycomb-like HUD network pattern. (c) Absorptance spectra measured for the Si membrane with (red) and without (blue) the HUD pattern with ARC. The membrane is suspended in air and infiltrated with a polymer resist (*n*_LRM_ = 1.52) as ARC. The dashed black line corresponds to the Lambertian limit absorption for 1 μm Si, based on the optical properties given in the literature.^55^

We demonstrate the power of hyperuniform disordered (HUD) patterns for lightweight, flexible, and efficient photovoltaics, by first focusing on the absorption properties in ultrathin (∼1 μm) Si. The proposed structure for the highly efficient Si light absorber is shown in Figure 1a. It consists of a thin Si slab (1 μm), of which the top 200 nm is patterned with an optimized HUD pattern. In this case, the pattern consists of a 2D network of Si walls, that resembles the honeycomb underlying structure in black butterfly wings.^29,56^ The Si pattern is infiltrated with a low refractive index medium by spin coating a polymer resist^57^ with refractive index of 1.52. While the infiltrated pattern layer is expected to also reduce reflectance due to the better index matching with air (*n*_pattern_ ≈ *n*_Si_·*f* + *n*_LRM_ (1 – *f*), with *f* being the Si filling fraction), an additional layer of resist on top (50–100 nm thick) further improves antireflectance, referred to as ARC. While the optical properties of the resist are not the ideal to guarantee minimal reflection, spin coating is a simple conformal fabrication method that ensures nanopattern filling and a flat top surface. Figure 1b is a photograph of a suspended 4.8 × 4.8 mm^2^ Si membrane nominally 1 μm-thick on a Si support frame before spin coating the resist. The membrane reveals a semitransparent reddish color owing to its small thickness and small absorption coefficient in the red and near-infrared. HUD-based patterns were fabricated on the membrane with e-beam lithography in at least 100 × 100 μm^2^ areas. These areas are clearly visible in the picture as they appear darker, highlighting the increased light trapping. A close-up scanning electron microscopy (SEM) image of the fabricated HUD network pattern on the Si suspended membrane is shown as inset.

We have measured the light absorption of the free-standing membrane with ARC on the unpatterned and patterned regions by using an integrating sphere microscope.^58,59^ The curves in Figure 1c compare the light absorption as measured for the flat membrane with that of the HUD patterned membrane. The absorption spectrum of the flat membrane shows the characteristic Fabry-Pérot interferences for a 1.18 μm thick Si slab (see Supporting Information), with a peak in absorption at about a wavelength of 450 nm and rapidly decreasing absorption for longer wavelengths due to the small absorption coefficient of Si in the visible and near-infrared. In contrast, the absorption in the membrane with the HUD pattern is on average 50% higher in absolute numbers for the wavelength range of 500 to 900 nm and it follows the Lambertian limit trend (4*n*^2^ limit for 1 μm-thick Si represented by the black dashed curve), which does not take into account reflection losses. Despite the fact that the membrane sits in air (i.e., no back-reflector) and the suboptimized ARC, the fraction of absorbed solar photons in the membrane increases from 25.5% to 66.5% by texturing the surface based on our optimized HUD design. This is the highest demonstrated absorption in a 1 μm Si absorber so far, and translates to a photocurrent of 26.3 mA/cm^2^, far above the 19.72 mA/cm^2^ in the best reported cell with similar thickness.^60^ Simulations show that a metal back reflector will increase absorption even further for the whole spectrum and integrated absorption can reach up to 93.4% of the Lambertian absorption. In the following, we describe the design principle and physical mechanism that induces this record in absorption.

### Light Trapping Mechanism and Design Optimization

**Figure 2:**
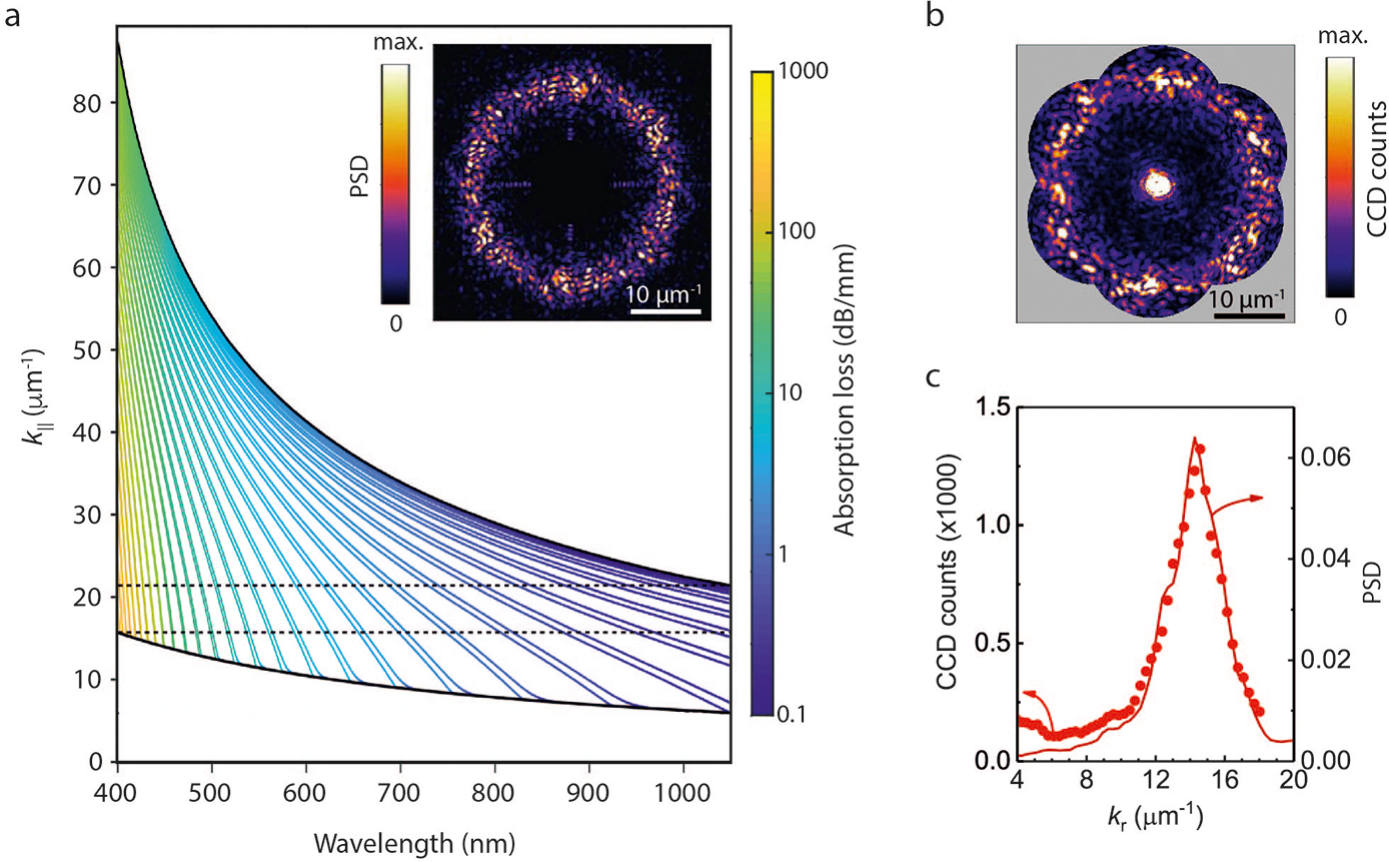
Light trapping mechanism. (a) Waveguide modes for a homogeneous 1 μm thick Si slab between air and a perfect metal for the wavelength range of interest in a solar cell. The modes are all lossy, with the absorption loss denoted by their color, as shown in the color bar. Black lines denote the dispersion curves for air (lower curve) and for Si (upper curve). The horizontal dashed lines denote two characteristic wavenumbers: *k*_1_ = 15.71, and *k*_2_ = 21.41 μm^–1^ as described in the text. Inset: Simulated power spectral density (PSD) of the HUD network design that shows a characteristic diffraction ring in the desired *k*-space. (b) Measured Fourier-space diffraction pattern in reflection of the *HUD network* design lithographically patterned in a Si wafer (wavelength 561 nm). (c) Radial distribution of the PSD in (a) and the diffraction intensity in (b).

As in previous works that use periodic and heuristic aperiodic structures to promote light trapping, the main mechanism by which the HUD nanostructure enhances absorption is diffraction into the absorber.^22,61,62^ In the presence of texturing, the guided modes of the thin silicon slab become leaky (quasi-guided) and can in- and out-couple to the incoming electromagnetic modes supported by the surrounding medium. The waveguide mode dispersion for a Si slab is shown in Figure 2a, where we note that within the spectral region of interest, c-Si exhibits strong dispersion that leads to a strong curvature of the Si light cone and significantly different absorption of the guided modes, as indicated by the color scale in Figure 2a. The total absorption is obtained by summing up the coupling contributions of each mode. To maximize sunlight absorption in the slab we need to couple efficiently to these lossy modes that exist for *k*_∥_ above the light-cone of air (lower black thick curve), for a broad range of wavelengths (from 350 to 1100 nm). Due to the large number of modes in a 1 μm Si slab, a pattern structure that diffracts incident light (*k*_∥_ = 0 for normal incidence) to the range of *k*_∥_ from ∼15 to ∼20 μm^–1^ (indicated by the two horizontal dashed lines in Figure 2a) ensures all sunlight has a mode to couple to. However, due to the inhomogeneous absorption of the guided modes, coupled-mode theory calculations estimate that the highest absorption is actually given for uniform diffraction to the *k*-range from ∼9 to ∼25 μm^–1^ (see the detailed calculation in the Supporting Information). Targeting this wave vector range is a key design goal for engineering the diffraction pattern.

In contrast to periodic and random patterns, hyperuniform designs with correlated disorder are an intermediate concept that allows the creation of diffraction into only a tailored range of wavevectors. In particular, stealthy HUD structures offer a distinctive route to filling desired bands in Fourier space, intermediate between the continuous *k*-space content of random patterns, and the discrete crystal momenta of periodic point patterns. Stealthy HUD point patterns are isotropic with no diffraction below a certain critical wavevector value, *k*_*C*_: *S*(*k*_∥_ ≤ *k*_*C*_) = 0. The so-called stealthiness parameter χ is defined as the fraction of wavevectors for which the structure factor vanishes and can be used to measure the hyperuniform correlations. Thus, χ = 0 for purely uncorrelated (Gaussian) point patterns and χ > 0.77 for periodic structures.^33,43,45,49^

Our design approach starts with a 2D HUD point pattern (χ ∼ 0.4–0.5) that provides the most uniform filling in the Fourier space domain delimited by the two wavenumbers (*k*_∥,1_ and *k*_∥,2_) estimated from the waveguide properties of the slab and coupled mode theory (see Supporting Information for more details). Once the 2D HUD point pattern is created, it is transformed into a physical 3D design that can be fabricated with two material components: Si and a low refractive index material. In this case, the 2D HUD point pattern is decorated with 200 nm tall Si walls following a Delaunay tesselation protocol^45^ that form a continuous Si network, and the voids are filled by the low refractive index material. However, light absorption with the 2-phase design is no longer expected to be optimal as the 3D texture strongly disrupts the waveguide properties of the Si slab. We note that upon the decoration of a 2D point pattern with a physical 3D unit cell geometry, the structure factor of the point pattern is no longer a suitable representative of the texture’s diffraction, due to the scattering properties of the unit cell. We therefore consider the power spectral density (PSD), which is the squared Fourier transform of the 2D design as a better representation of scattering strength. In the first Born-approximation, i.e., the limit of single scattering that applies to low index-contrast structures, this corresponds to evaluating the product of structure factor (Fourier transform of the lattice geometry) and form factor (single unit cell scattering function approximated as Fourier transform of the geometry). This approach neglects multiple scattering and coupling effects, and is a fair approximation in the low index limit^63−65^ The tesselation protocol thus causes the resulting 3D network to become nearly hyperuniform as it slightly deviates from hyperuniformity constraint^66^ (its PSD may display a small but nonvanishing amplitude for *k*_∥_ < *k*_*c*_).

To resolve this nonideality, we introduce a second optimization step to fine-tune the HUD-based 3D pattern, where the HUD properties (average distance between points) and Si filling fraction are optimized. This is done with full-wave 3D numerical simulations that compute light absorption at each optimization step (see Materials and Methods and Supporting Information). While the process is computationally expensive, the initial 2D HUD optimization procedure sets an excellent base to rapidly find a local maximum. As a result, we obtain an optimized 3D structure based on a 2D HUD point pattern, but with a PSD function that may differ from what was initially estimated from the structure factor of the point pattern and slab waveguiding properties. The inset in Figure 2a shows the 2D simulated PSD of the fully optimized HUD network design that was used to create the sample in Figure 1. The PSD for the first order diffraction shows a clear fingerprint of the hyperuniformity with a circular region around *k*_∥_ = 0 where *S*(*k*) vanishes, but there is not a sharp cutoff as initially imposed. The nearly hyperuniform PSD is better discerned in the angle-averaged PSD (solid curve) shown in Figure 2c. Notice that the Fourier space in the optimized pattern is filled in the wavevector region between 10 and 18 μm^–1^, which is slightly different than the initial guess for *k*_∥,1_ and *k*_∥,2_ of 9.7 and 20.4 μm^–1^.

We have performed momentum spectroscopy of the fabricated pattern on a Si surface, where the measured *k*-space diffraction pattern in reflection is shown in Figure 2b as obtained using high-NA Fourier microscopy.^67^ By construction the HUD pattern is designed to scatter normally incident light to parallel wave vectors that are *outside* the collection NA of air objectives. However, by combining strongly off normal illumination at six azimuthal angles we can reconstruct the parallel-momentum resolved scattering properties up to an effective NA almost twice higher than that of the objective lens (see full details in the Materials and Methods section). The angle-resolved diffraction measured in reflection displays a similar fingerprint of the hyperuniformity as the 2D PSD of the design. The measured angle-averaged reflection is also plotted in Figure 2c, and is extremely well reproduced by the theoretical PSD (solid line).

### Comparison of HUD-Based Designs

**Figure 3:**
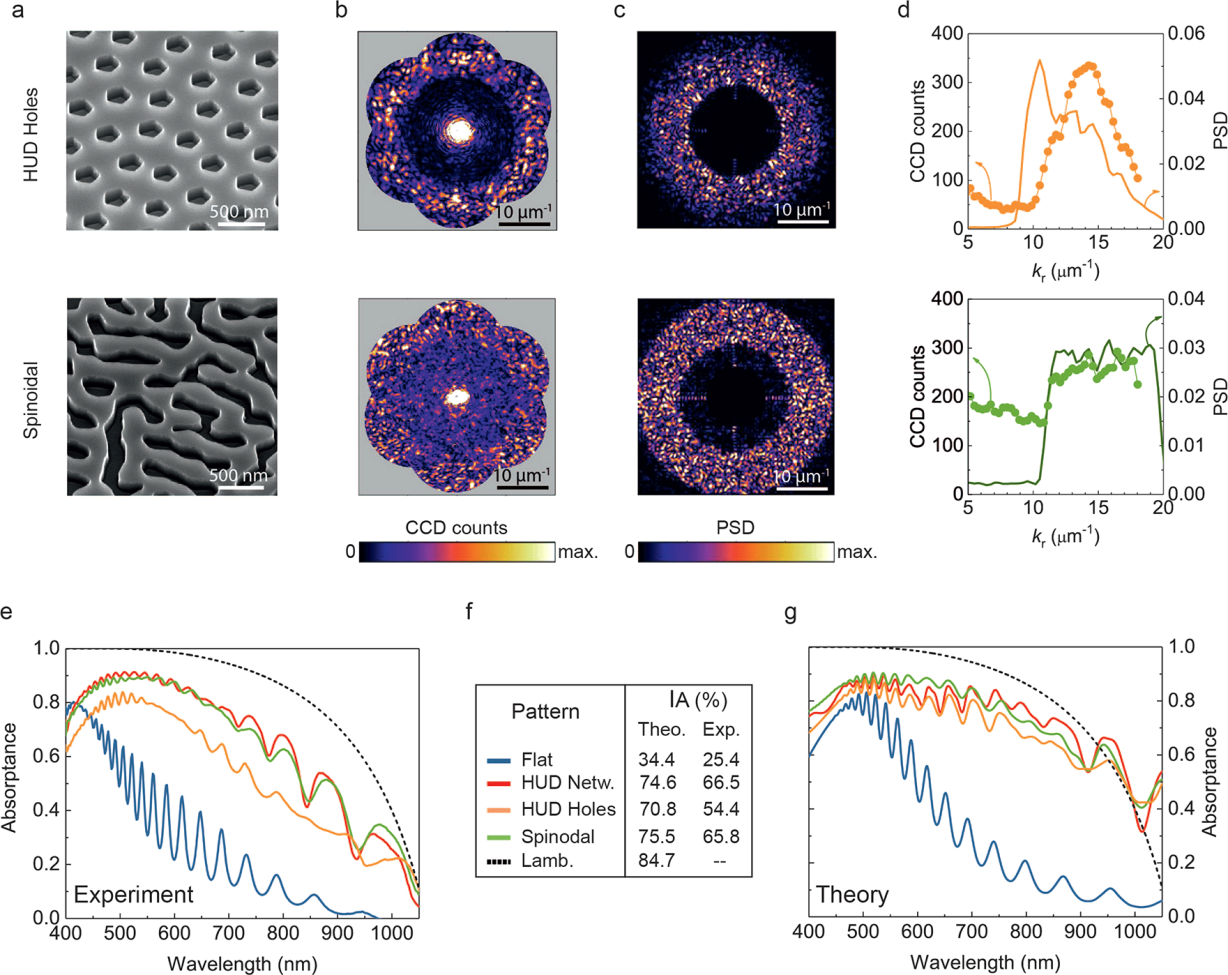
Performance comparison between different HUD-based designs. (a) SEM images of samples textured with the *HUD hole* (top) and *spinodal* (bottom) designs. (b) Measured angle-resolved diffraction in reflection of the corresponding pattern. (c) Simulated 2D PSD for the optimized *spinodal* and *HUD hole* patterns. (d) Diffraction intensity as a function of in-plane wavevector (*k*_*r*_) given by the angle-averaged simulated PSD (solid curve) and measured diffraction (dots). Measured (e) and calculated (g) absorptance spectra for a 1 μm thick Si slab suspended in air with the different surface nanopattern designs with ARC considered. The absorption spectrum for the *HUD network* design is the same as in Figure 1. The theoretical Lambertian limit and the absorption for a flat Si slab (with an ARC) are shown as reference. (f) Table listing the percentage of integrated absorbed solar photons (IA) for all patterns for the wavelength range of 400–1050 nm. These numbers are obtained by integrating the theoretical or experimental absorption spectra over the solar flux (AM1.5G) and normalizing for the total photon flux in the specified wavelength range.

So far, we have shown that a 2D HUD point pattern can lead to a highly efficient 3D design for broadband light trapping in a thin Si slab, by *decorating* the point pattern with two materials in a wall network fashion (Figure 1b). However, there are many other decorating possibilities for the same initial 2D HUD pattern. For instance, instead of the wall network, one could place Si nanopillars or nanoholes at the points of the 2D HUD point pattern and fill the voids with ARC. This is the simplest HUD design, where a single element is cloned at tailored positions. We refer to this texture as *HUD hole*, and the SEM image of the as-fabricated sample is shown in Figure 3a. Similar to the *HUD network* in Figure 1, the Fourier microscopy intensity map, Figure 3(b, top), indicates the HUD nature of the design and it is very similar to the theoretical structure factor, Figure 3(c, top).

Another very different way of obtaining 3D HUD patterns is inspired by the generation of *spinodal* structures.^68,69^ Here the mathematical recipe is to first define a random superposition of cosine waves with random phase, with wave vectors imposed by the desired wave vector distribution (*k*_∥,1_ ≤ *k*_∥_ ≤ *k*_∥,2_), or structure factor. Thresholding the resulting function at a fixed height value defines material boundaries separating Si and low refractive index material (filling fraction *f* set by threshold choice), tracing out zebra-like patterns as in Figure 3(a, bottom). The resulting two-phase material pattern is nearly hyperuniform, for which its PSD is dominated by the wave vector distribution imposed at the initial design stage. Owing to its inspiration, we refer to this design as *spinodal*. Fourier microscopy of the as-fabricated *spinodal* design, Figure 3(b, bottom), also exhibits a characteristic low scattering at small wavevectors, and a marked increase of scattering at *k*_∥_ ∼ 11 μm^–1^. The contrast is lower compared to the other HUD designs and the scattering at small wavevectors is not expected from the theoretical PSD, Figure 3(c, bottom). We suspect the additional scattering at low wavevectors arises from fabrication imperfections due to the sensitivity of the PSD of spinodal design to the Si filling fraction (see Supporting Information).

The azimuthally averaged *k*-space resolved diffraction and theoretical PSD for the *HUD holes* and *spinodal* designs are shown in Figure 3d as dots and solid lines, respectively. While the PSD of the *HUD holes* is similar in shape to that of the *HUD network* pattern with a peak at ≈15 μm^–1^, the PSD of the *spinodal* is quite different and resembles a square function. All three proposed designs have a PSD that is well suited to couple normally incident sunlight into the plane for absorption. The theoretical and experimental absorption spectra for the patterned and unpatterned suspended membranes are shown in Figure 3e and g, where the ARC (same parameters for all designs) is also taken into account. The spectrum for the *HUD network* pattern is also included and it is the same as in Figure 1c. As a quantitative measure to compare absorption between all the different designs, we have computed the fraction of absorbed solar photons (integrated absorption, IA), as listed in the table in Figure 3f. The IA, is computed by considering the AM1.5G solar spectrum for the wavelength range of 400 to 1050 nm (see the Materials and Methods section for more details). For comparison, we also plot the Lambertian limit obtained by considering the same optical constants used in the full-wave simulations. Similar to the spectra measured in the *HUD network* patterned membrane (red curve), the two new designs raise light absorption in the long wavelength regime, particularly in the case of the *spinodal* (green curve). As expected from the absorption spectra, the *spinodal* and *HUD network* patterns result in a similar IA (∼66% in practice and ∼75% in theory). As also predicted by theory, the *HUD holes* design leads to a slightly lower absorption and IA (∼54% in practice and ∼70% in theory). Part of the discrepancy between theoretical and experimental IA may be attributed to the overestimation of the extinction coefficient of Si in the NIR (up to 4% absolute, equivalent to up to 1.6 mA/cm^2^, see Supporting Information for more details). We attribute rest of the discrepancy between theory and experiment (4–6% absolute IA, equivalent to 1.6–2.4 mA/cm^2^) to the nonideal scattering from fabrication imperfections, local deviations in the ARC and membrane thickness, as well as the pattern design being optimized with an overestimated extinction coefficient of Si in the NIR. In any case, for all three designs the measured IA is more than twice that of the unpatterned membrane and we experimentally demonstrate for the first time and for two patterns that absorption in a free-standing Si membrane is as high as ∼78% of the Lambertian limit.

### Full Device Design and Efficiency Estimation

**Figure 4:**
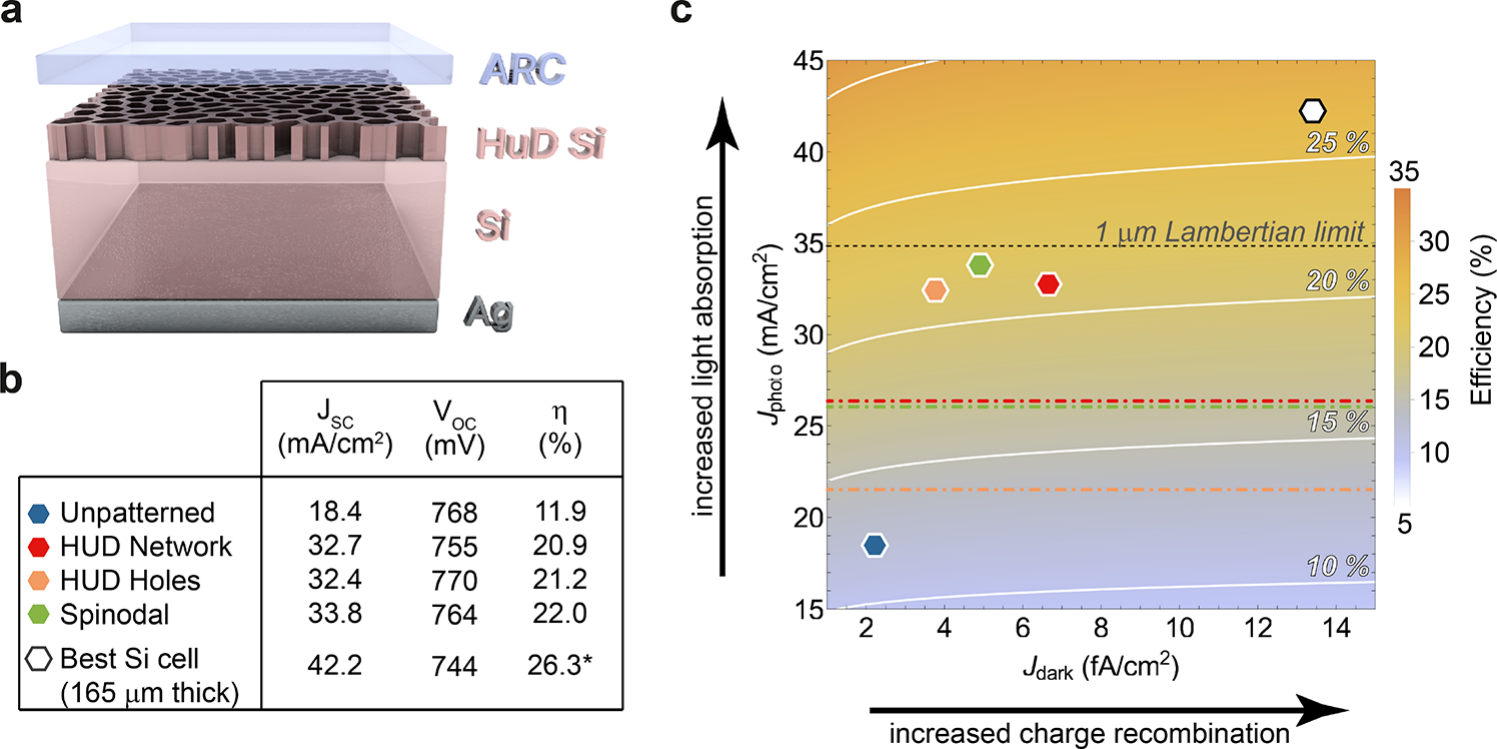
PV efficiency estimation. (a) Full solar cell device design, which includes a Ag back-contact and improved ARC (*n*_LRM_ = 1.82 and 72 nm thick). (b) Table summarizing the estimated PV performance parameters from our optical and PC1D device simulations. (c) Color-map indicating the nonlinear dependence of the maximum PV efficiency on the dark and photocurrents (*J*_dark_ and *J*_photo_, respectively). The white lines are isolines at the efficiency indicated by the labels. The dashed black line corresponds to the *J*_photo_ given by the Lambertian limit in a 1 μm-thick Si slab. The dash-dotted lines correspond to the *J*_photo_ derived from our absorption measurements without back-reflector. The data points correspond to the estimated PC1D efficiencies for the different designs. The efficiency estimated for the unpatterned Si membrane and that for the best demonstrated bulk Si cell are shown for comparison. The total efficiency is not only affected by increased light trapping, but also by the additional pattern-induced surface area recombination, reflected in the increased dark current.

So far, we have demonstrated the exceptional light trapping properties of the HUD patterns in thin Si, as evidenced by the enhanced absorption. However, in a full solar cell device one must also consider other effects of nanotexturing on its performance. It is important to ensure that the gain from light trapping for PV remains despite the potential penalty of increased surface recombination, which can strongly affect the performance of devices with a Si thickness smaller than ∼90 μm, where bulk-related losses are negligible.^5,6^ In order to understand the effects of our HUD-based designs on the PV efficiency, we consider the full device structure shown in Figure 4a. Now, the patterned Si film sits on top of a silver metal contact that also acts as a back reflector. By using interdigitated macroscopic Ag pads, both the *n* and *p* contacts are placed at the rear, which reduces shading on the front of the cell. This technology, known as interdigitated back contact (IBC) photovoltaics, has enabled the highest PV efficiency in Si-based cells.^70−72^ For simplicity, in our absorption calculations we consider a continuous Ag film at the back instead of the interdigitated pads, which is a fair assumption for optical purposes given their large characteristic sizes.

We have reoptimized the 3D pattern design taking into account the Ag back-reflector as part of the full structure. We also consider an improved ARC configuration, with *n*_LRM_ = 1.82 and ARC thickness of 72 nm (see Supporting Information). The resulting absorption spectra (for the Si layer only) for all the different designs are shown in the Supporting Information. As expected, the metal back-reflector and improved antireflection increases light absorption as compared to that shown in Figure 3g. Note that these account for absorption in Si only, where parasitic absorption in the metal would increase the total absorption in the device stack by an additional 10% in average for wavelengths 600 nm and above. Interestingly, the three designs offer a highly robust absorption to changes in the angle of incoming light (see the angle dependent absorption spectra in the Supporting Information), which strongly enhances the daily PV power output.

We have computed the photocurrent, *J*_photo_, by integrating the simulated absorbed solar spectrum for the wavelength range of 300 to 1050 nm. To estimate the PV performance of our designs we have simulated the current–voltage characteristics of the solar cell with the PC1D software, where we have considered *J*_photo_ as input and a bulk lifetime of 0.5 ms (standard PV grade Si) and surface recombination velocity (SRV) of 100 cm/s. Given that the absorber thickness is much smaller than the diffusion length for minority carriers in Si, the 1D approximation used by the PC1D is valid to account for losses in the short-circuit current. The SRV value of 100 cm/s is the state-of-the-art in high efficiency Si solar cell devices.^1,70^ In order to account for the pattern-induced increased surface area, we have considered an effective SRV by multiplying it by the surface area increase factor. A more detailed description of the parameters used in our simulations is given in the Supporting Information. The table in Figure 4b summarizes the estimated solar cell performance, in terms of short circuit current (*J*_SC_), open circuit potential (*V*_OC_), and PV efficiency (η), for the optimized *HUD hole*, *HUD network*, and *spinodal* patterns. For comparison, we have included the theoretical case of an unpatterned Si membrane (with ARC) and the current record Si cell, which is 165 μm thick.^70^

As explained in previous works, the surface recombination velocity has a minor effect on the short circuit current as the cell thickness is of the order of the carrier diffusion length^6,12,73^ and thus the photocurrent is very close to the integrated absorption (*J*_photo_ ∼ *J*_abs_). Additionally, we assume no external resistance losses, thus *J*_SC_ = *J*_photo_. With the pattern-induced light trapping, the *J*_SC_ in 1 μm-thick Si is almost doubled for all three designs compared to the unpatterned cell (from 18.5 mA/cm^2^ to 33 mA/cm^2^), close to the Lambertian value for 1 μm-thick Si (at 35 mA/cm^2^). At the same time, the estimated *V*_OC_ values in our three designs are higher than the best bulk cell and oscillate around that expected in an unpatterned thin film. Because of the small volume in a 1 μm film compared to bulk, the saturated dark current, *J*_dark_, in the cell is only limited by surface recombination. Considering the state-of-the art bulk carrier lifetime and surface recombination velocity, *J*_dark_ is almost 1 order of magnitude smaller in a 1 μm Si film compared to Si bulk (165 μm thick) and thus the *V*_OC_ is improved by thinning Si down.^1,5,12,74^ An interesting consequence of this is that lower grade Si material can thus be used in such thin devices, which have much lower costs (see the Supporting Information for more details on how the PV efficiency is affected by the bulk lifetime and surface recombination). As *V*_OC_ also depends on the photocurrent, *J*_photo_, light trapping has the potential to compensate the effects of patterning-induced larger *J*_dark_ on the voltage. We find that the *HUD holes* design uses the smallest surface area and the strong light trapping fully compensates the effect of increased surface recombination and leads to the same *V*_OC_ as in the unpatterned case.

In terms of efficiency, Figure 4c represents the two major consequences of nanotexturing, increased light trapping and increased charge recombination, on the PV efficiency (color scale) by considering the illuminated diode equation (*J*(*V*) = *J*_dark_(*e*^*qV*/*k*_B_*T*^ – 1) – *J*_photo_). The dashed horizontal black line corresponds to the ultimate photocurrent from the Lambertian light trapping in 1 μm thick Si and one can see the span of possible PV efficiencies depending on the carrier recombination properties of the device. For fair comparison to current densities in the optimized devices, we have used here the same Si optical constants as in the simulations, which leads to an overestimation of the photocurrent of 1.7 mA/cm^2^ (equivalent to maximum of 1% overestimation in the conversion efficiency). Similarly, the colored dashed-dotted lines indicate the photocurrent based on the measured absorption in the patterned free-standing membrane. For the integration boundaries from 400 to 1050 nm (i.e., measured spectral range), we obtain photocurrent values of 21.5, 26.3, and 26.0 mA/cm^2^ for the HUD holes, HUD network, and spinodal, respectively, which are the highest demonstrated so far in <10 μm-thick Si.^75^ It is interesting to note that the strong light trapping power the HUD network and spinodal patterns measured in our membranes could result to cell efficiencies above 15%.

In the graph, we also include data points for the unpatterned and the three optimized HUD cell designs (i.e., with metal back reflector), based on the *J*_photo_ and *J*_dark_ values expected from our light absorption simulations and from the advanced IBC and surface passivation technologies available today, respectively. For comparison, we also include the data point for the best demonstrated Si solar cell, which is 165 μm thick.^70^ Note that the diode equation yields a slightly higher PV efficiency compared to that reported in ref (70) (0.5% higher efficiency) as the model neglects contact resistance losses.

Figure 4c clearly visualizes the compromise for the total efficiency between light trapping and surface recombination properties of the different designs and it is particularly evidenced by the *HUD holes* and *HUD network*. Despite the fact that the two designs have different light trapping capabilities, the final PV efficiency is very similar. It is interesting to note that even if the best surface passivation is not achieved in practice, the large absorption and thin device configuration allows for efficiencies above 20% for a large range of dark current. This is given by shifting the data points in Figure 4c to larger dark current densities, which will still be above the 20% isoefficiency line.

While we theoretically predict the ultimate best device to be that with the *spinodal* texture, the large scale implementation of its fabrication may require some further technological developments. A combination of interference and nanoimprint lithography^76^ has already demonstrated the fabrication of aperiodic structures with defined spatial frequency distribution in areas larger than 1 m^2^. However, further work is needed to increase the required patterning resolution down to few tenths of nanometers. By contrast, HUD point patterns naturally arise in many physical systems, which can lead to simple and scalable fabrication of the *HUD holes* or *HUD network* patterns. For instance, it has been shown that the structure factor of dispersed colloidal particle (e.g., beads) patterns can be tuned by the ionic strength of the particular solvent^77−80^ and that the dewetting or phase separation of dielectric layers leads to HUD patterns.^81,82^ Also, soft-imprint conformal lithography has proven an excellent low-cost alternative to pattern large-areas with a resolution below 10 nm that could actually serve for both *HUD holes and HUD network*.^83^ While a master substrate has to be first made with other lithography methods (such as e-beam lithography), the master can be extensively reused for the creation of multiple-use soft stamps.

## Conclusion

We have shown that stealthy HUD point patterns are an excellent platform to design a wealth of highly efficient nanoscale textures for trapping light in ultrathin Si solar cells. We have described three different texture designs that offer broadband isotropic light trapping with a characteristic hyperuniform signature in the Fourier reflectance. We have fabricated such textures on a suspended Si membrane and experimentally demonstrated the highest absorption in 1 μm-thick Si, corresponding to a *J*_photo_ of 26.3 mA/cm^2^. This exceptional light trapping can potentially be further improved by optimizing the antireflection coating and incorporating a metal back-reflector, which in turn serves as electrical contact. Taking into account state-of-the-art values of Si passivation and IBC device design, we estimate that PV efficiencies above 20% could be achieved for a 1 μm-thick c-Si cell, which represents a breakthrough toward flexible, lightweight c-Si PV.

## Materials and Methods

### Generation of the Disordered Hyperuniform Structures

A hyperuniform point pattern is a random point pattern in real space for which the number variance σ^2^(*R*) within a spherical sampling window of radius *R* (in *d* dimensions) grows more slowly than the window volume (∝*R*^*d*^) for large *R*. We consider point patterns that are stealthy, which is a property of the structure factor *S*(**k**), defined as^32^
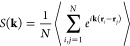
1where **k** are vectors in reciprocal space, *N* is the total number of particles, **r**_*i*,*j*_ are the positions *i*-th and *j*-th particles, and ⟨...⟩ denotes ensemble average. In all subsequent analysis we omit the forward scattering contribution, i.e., consider the *S̃*(**k**) = *S*(**k**) – (2π)^*d*^ δ(**k**) quantity, with *d* the dimensionality of point distribution. For stealthy point patterns, *S̃*(**k**) is isotropic and vanishes for a finite range of wave numbers 0 < *k* ≤ *k*_0_, for some positive critical wave vector, *k*_0_.^34^ The size of this region can be expressed through the so-called stealthy parameter χ = *M*(**k**)/*dN*, where *M*(**k**) is the number of linearly independent **k** vectors where *S*(**k**) = 0 and *d* = 2 in the present case.^34,45^

### Generation of the Spinodal Pattern

Density wave (spinodal) structures or spinodal structures are a particular realization of hyperuniform structuring and can be generated according to a simple protocol.^68^ We consider the function
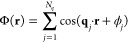
2where *k*_1_ < |**q**| < *k*_2_ is a collection of homogeneously distributed random *N*_*q*_ wavevectors, and ϕ_*j*_ are random phases uniformly distributed in the range (0, 2π). This function is hyperuniform by construction with its Fourier transform uniformly distributed in the *k*-space ring defined by *k*_1_ < |**q**| < *k*_2_. To obtain a two-phase dielectric function we define
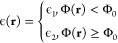
3where ϵ_1_ and ϵ_2_ are relevant dielectric permittivities of the two phases, and Φ_0_ is a value chosen to yield the desired filling fraction. We note the cut procedure defined above may violate the strict hyperuniformity constraints, and in practice the resulting structures are nearly hyperuniform with most of the Fourier components concentrated in the *k*-space ring defined by *k*_1_ < |**q**| < *k*_2_.

### Power Spectral Density

In this work we use both the structure factor *S*(**k**) and the power spectral density or PSD. As explained above, the structure factor *S*(**k**) is used as generation seed. Instead, we use the PSD as an informative quantifier for structure performance and for experimental data comparisons (to single-realization 2D diffraction patterns) as PSDs account also for the single-unit cell scattering properties. The structure factor is an ensemble-averaged property of point patterns, meaning that (1) *S*(**k**) takes into account solely the distribution of particle sites, and not the single-particle scattering properties, and (2) it is a statistically averaged property (⟨...⟩, averaging over many realizations). Instead, the PSD gives the scattering properties for a single structure realization and including the scattering properties of the single unit cell, under the assumption that the first Born approximation applies. For a binary spatial distribution of dielectric material ϵ(**r**) = ϵ_1_ + (ϵ_2_ – *e*ϵ_1_)*f*(**r**) the relevant PSD reads PSD = |∫ *f*(**r**) *e*^*i***k**·**r**^*d*^2^**r**|^2^. We note that for collections of identical particles located at a set of points *r*_*j*_, the function *f*(*r*) is the convolution of a particle shape with a point set, so that by the convolution theorem, the PSD is the product of the Fourier transform of the lattice, and the Fourier transform of the single particle shape. Physically, this recovers the well-known statement that in the first Born approximation the angle-resolved array scattering is given as the product of the single-unit cell scattering function (form factor), and the Fourier transform of the aperiodic lattice. We refer to refs (64, 78, 79, 84).

### Absorption Simulations

Optical simulations were performed using a freely available finite-difference time-domain (FDTD) solver.^85^ In all cases, the disordered structures were generated under periodic boundary conditions and are modeled as supercells with sizes between 10–15 μm. For absorption simulations, we have employed periodic boundary conditions in the transverse directions and perfect matching layer boundary conditions in the longitudinal direction. The dispersive dielectric function of Si was modeled using a sum of Lorentzian terms^28^ as detailed in the Supporting Information and the mesh resolution was 5.2 nm. To calculate the absorption, the structure was illuminated by broad bandwidth plane waves pulses, and the subsequent transmitted and reflected fluxes were recorded for a long simulation time. For the reflectance and transmittance of the Si layer, we have employed a reflection monitor at the top of the structure and a transmission monitor between the silicon and metal layer, respectively. Due to the diffusive character of the wave propagation and presence of various localized resonances in the disordered texture layer, the simulation is not run for a fixed amount of time but it keeps running until the field in the slab have decayed by a factor of 5 × 10^–5^ from its peak value in an interval of 20 simulation time units.^85^

### Sample Fabrication

Single crystal ⟨100⟩ 1 μm Si membranes (Norcada Inc.) were used. The actual thickness of the membrane may vary slightly. From the Fabry-Pérot interference pattern in the absorption spectrum for the unpatterned membrane nearby the patterned areas, we deduce a total thickness of 1180 nm. The membranes were either 1.3 × 1.3 mm^2^ or 4.8 × 4.8 mm^2^ in size in a Si frame of 10 × 10 mm^2^ and 300 μm thick. The nanopatterns were made by electron beam lithography followed by reactive ion etching. First, CSAR e-beam resist was spin coated as mask. Fields of either 100 × 100, 150 × 150, or 180 × 180 μm^2^ patterns were exposed. After exposure and development, 200 nm of the Si membrane was etched by first removing the native oxide followed by HBr and O_2_ etching. The left-over resist was lifted off, and the sample was ready for Fourier microscopy. For the absorption measurements, an additional layer of resist (OrmoComp resist) was spin coated on top of the sample to act as antireflective coating, ARC. The effects of the ARC on light absorption are described in the Supporting Information. From the interference fringes in the absorption spectrum taken on the unpatterned area, we deduce that the resist layer is of 200 nm.

### Fourier-Space Illumination and Imaging

In order to experimentally characterize the structure factor of the hyperuniform structures, we employed high-NA back focal plane imaging, also known as Fourier microscopy. In this technique angle-dependent scattering patterns of a sample are captured in single shot measurements, as opposed to performing angular scans using a rotation stage. We used a home-built inverted microscope (reported in ref (67)) that operates in reflection mode. To prevent reflections from the back surface we probed the designs fabricated onto a standard Si wafer. Also, the membrane is easily broken when retrieving the sample in the inverted microscope, due to statics. The microscope is infinity corrected with an Olympus MPlan IR 100× NA = 0.95 objective, a 200 mm tube lens and 200 mm Fourier lens.

As the light source we use a cw DPSS laser (Lasos DPSS) with a wavelength of 561.3 nm. At this wavelength, the actual microscope NA equals 0.89, as calibrated with a diffraction grating. The image of the objective back focal plane is relayed to an Andor Clara silicon CCD camera. According to the Abbe sine condition, captured Fourier images directly map parallel momentum space, as scattering at angle θ, ϕ (polar angle relative to sample normal and azimuthal angle, respectively) projects onto the camera plane at location (*x*, *y*) = *f*_o_(cos ϕ sin θ, sin ϕ sin θ) ∝ **k**_∥_, where *f*_o_ is the microscope objective focal length (*f* = 1.8 mm). Since we essentially measure the structure factor as a function of *k*_∥_ (wave vector parallel to the Si/air interface), one would expect to see the same dependence in both reflection and transmission.

Essential to our experiment is that the HUD patterns have structure factor *S*(**k**_∥_) predominantly at parallel momentum *just outside* the NA of our collection objective, corresponding to scattering normally incident light into guided modes. The ring of diffraction intensity distribution in momentum space follows *I*(**k**_∥_) = *S*(**k**_∥_ – **k**_in,∥_) and is therefore centered on the incident parallel momentum **k**_in,∥_. From free space one can therefore access the structure factor *S*(**k**_∥_) for parallel momenta up to *twice* the microscope NA by illuminating at multiple oblique incidence angles [].

In order to access any excitation angle without physically moving parts of the set up, the excitation path is equipped with a spatial light modulator (Meadowlark 1920 × 1152 XY Phase Series SLM) that is imaged onto the microscope back focal plane. By displaying regions of blazed phase gratings on the SLM, light can be selectively send to the first grating order. This allows an effective amplitude modulation, by placing an iris in the Fourier plane of the SLM, which blocks all light except for the modulated first diffraction order.^86^ By displaying a single small circle on the SLM and choosing its position, illumination with arbitrary **k**_in,∥_ can be generated. For each structure we collected six images arranged as the vertices of a hexagon. To obtain *S*(**k**_∥_), collected images were shifted by their respective *k*_in,∥_, while overlapping image areas were averaged.

### Absorption Measurements

Absorption measurements on the membrane were done by using an integrating sphere microscope (modified LabSphere GPS-020-SL with the 17 mm working distance objective Mitutoyo M Apo Plan NIR 50× and NA = 0.42) coupled with a supercontinuum laser (Fianium WL-SC-390-3) and an acousto-optical tunable filter (AOTF, Crystal Technologies, with roughly 4 nm bandwidth). The backscattered and transmitted light are both detected together and by adding the specularly reflected signal, we determine absorption. The photodetectors are Thorlabs amplified Si detectors (PDA100A), read out by Stanford Research Systems SR830 lock-in amplifiers. More details about the integrating sphere microscope setup can be found in ref (59). We have used three photodetectors to measure the reference beam, the reflected and the transmitted/forward scattered light, respectively. The light reflected back into the objective is detected with the reflection detector, while the integrating sphere detector detects the transmitted and scattered light. The absorbance is calculated by subtracting the reflected and transmitted/scattered power from the incident power. Two reference measurements in reflection and transmission were done with a glass slide and calibrated mirror, to account for the response function of the setup.

## Supporting Information Available

Design parameters; Absorption spectra in the full device; Silicon dispersion model; Mode coupling analysis; Angular dependence of absorption; Power spectral density robustness to fabrication imperfections; PC1D simulation details; Bulk lifetime and surface recombination effects; Integrating sphere setup (PDF)
