# Supplementary material for: Over 65% Sunlight Absorption in a 1 μm Si Slab with Hyperuniform Texture
Source: ACS Photonics. 2022 Mar 22;9(4):1206–17. doi: 10.1021/acsphotonics.1c01668 (PMC9026274; doi:10.1021/acsphotonics.1c01668)
Supplement: Supplementary file 1 — ph1c01668_si_001.pdf [file ph1c01668_si_001.pdf]

Supplementary Information
Over 65% sunlight absorption in a 1 μm Si slab with hyperuniform texture

Nasim Tavakoli,[†] Richard Spalding,[‡] Alexander Lambertz,[†] Pepijn Koppejan,[†] Georgios Gkantzounis,[‡] Chenglong Wan,[‡] Ruslan Röhrich,[¶,§] Evgenia Kontoleta,[†] A. Femius Koenderink,[†] Riccardo Sapienza,[‖] Marian Florescu,[*,‡] and Esther Alarcon-Llado[*,†]

†*Center for Nanophotonics, AMOLF, Science Park 104, 1098XG, Amsterdam, The Netherlands*

‡*Department of Physics, Advanced Technology Institute, University of Surrey, GU2 7XH, Guildford, United Kingdom*

¶*Center for Nanophotonics, AMOLF, Science Park 104, NL1098XG, Amsterdam, The Netherlands*

§*Advanced Research Center for Nanolithography, Science Park 106, 1098XG, Amsterdam, The Netherlands*

‖*The Blackett Laboratory, Department of Physics, Imperial College London, London SW7 2BW, United Kingdom*

E-mail: m.florescu@surrey.ac.uk; e.alarconllado@amolf.nl,+31(0)207547100

Design parameters and optimisation flow

Figure S1 shows a schematic representation of the optimisation procedure used in this work to achieve the final 3D HUD-based textures. We start with an empty canvas in k-space. Employing the properties of the Si slab waveguide modes and coupled mode theory (see section Mode Coupling Analysis) we infer a near-optimal diffraction k-range that maximises the absorption efficiency of the incoming solar radiation.

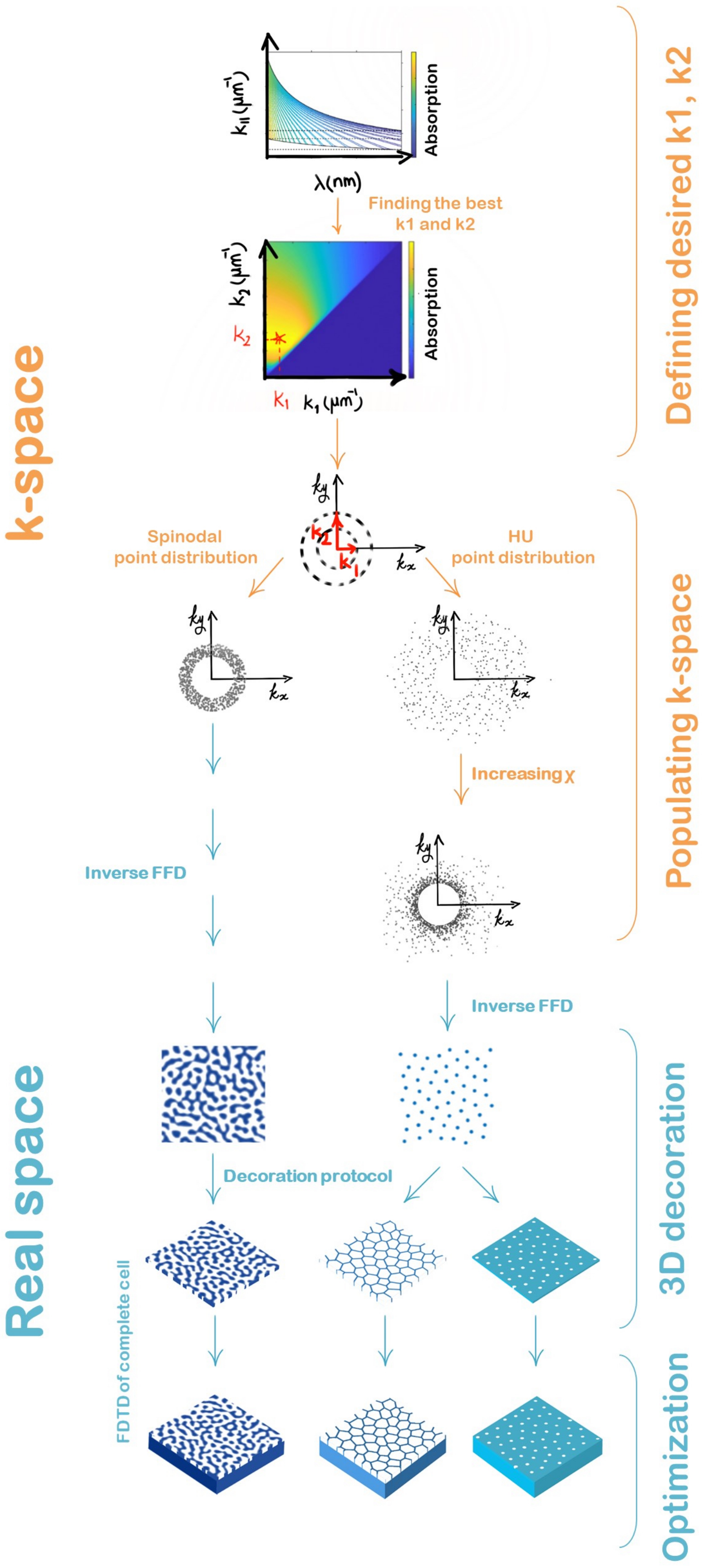

Fig. S1 Schematic representation of the optimisation flow, starting from top to bottom.

In a second step, we populate the k-space range uniformly based on the constraints derived in the previous step. For the HUD patterns, only the inner bound for the wavevector is used. As the stealthiness parameter χ is increased, a ring distribution naturally forms. Next. we identify the 2D point pattern is obtained whose Fourier transform reproduces the k-space pattern targeted. From the 2D point pattern hence generated, a 2D two-phase structure is obtained in a subsequent decoration step. For the spinodal patterns, the two-phase structure is obtained by the random superposition of cosine waves with random phase with wave vectors imposed by the k-space distribution and thresholding the resulting function at a fixed height value. As a final step, the two-phase pattern is extruded to a height h_G and incorporated as part of the full 3D solar cell. Figure S2 is a cross-section representation of the 3D device design that is considered for the FDTD calculations in the last optimisation step. The sames values for h_{AR}, h_G and n_{AR} have been fixed to all design. We have used the values as obtained from optimising light absorption in a Si slab with a periodic hexagonal pattern (the periodicity of which was also optimised).

In the final optimisation step of the HUD and spinodal patterned cells, light absorption in the film is calculated at each optimisation step where the average lattice spacing (a) and Si filling fraction (f) of the patterns is fine tuned. The optimised design parameter values are shown in Table S1.

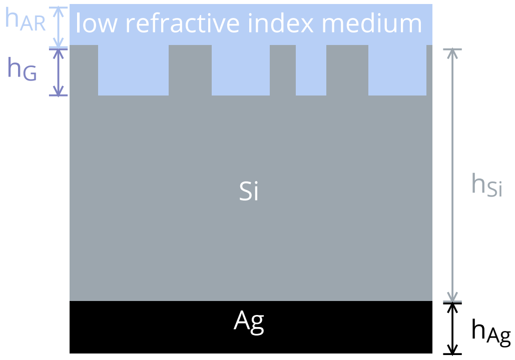

Fig. S2 Schematic picture of the device design

Experimentally, a polymer resist was used as the low refractive index material by spin coating and a commercial membrane with nominal thickness of 1 μm. From the interference

Table S1 Parameters for the structures optimised for the full device.

Pattern	h_{AR} (nm)	h_G (nm)	n_{AR}	f (%)	a (nm)	χ
Unpatterned	72	196	1.82	–	–	–
Periodic hex	72	196	1.82	79	644	–
HUD network	72	196	1.82	55	475	0.5
HUD holes	72	196	1.82	68	480	0.4
Spinodal	72	196	1.82	58	–	–

pattern in the absorption spectrum of the unpatterned membrane (Figure S3), we deduce the actual thickness of the membrane and resist (listed in Table S2). On the pattern, we expect the ARC layer to deviate from the resist thickness on the unpatterned region.

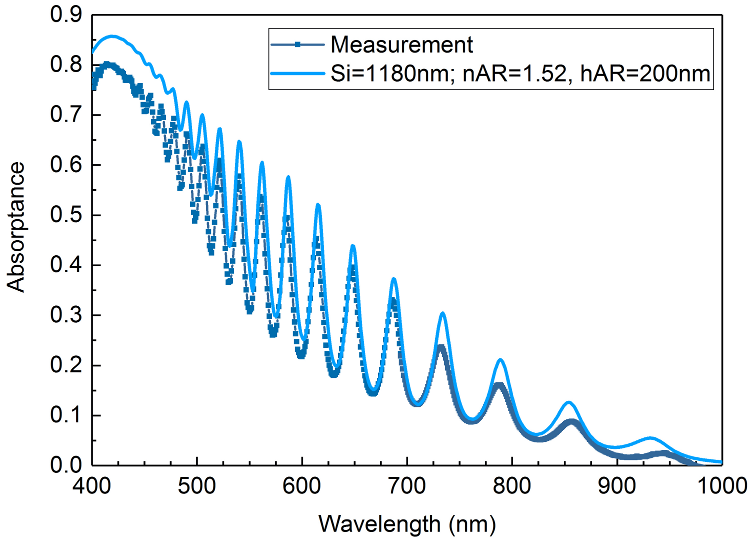

Fig. S3 Measured and calculated absorption for a Si membrane suspended in air with a top layer of refractive index 1.52.

Table S2 Values of the parameters for the fabricated and optimal structures. We note that the thickness of the Ag back-reflector has not been rigorously optimised but chosen to be thick enough to enable high reflectance.

Parameter	Fabricated value	Optimal value
h_{AR}	50-100 nm	72 nm
h_G	200 nm	196 nm
h_{Ag}	–	200 nm
h_{Si}	1180 nm	1000 nm
n_{AR}	1.52	1.82

Figure S4 is the calculated reflectance from a representative double layer anti-reflective coating on c-Si. The two layers consist of the infiltrated pattern (with an effective refractive index considering the filling fraction of Si for each pattern) and the ARC layer. By using the parameters listed above for ARC refractive index and thickness, pattern thickness and Si filling fraction and the double-layer ARC model described in Ref. 1, we find that reflectance is below 10% for the wavelength range of 400 to 900 nm.

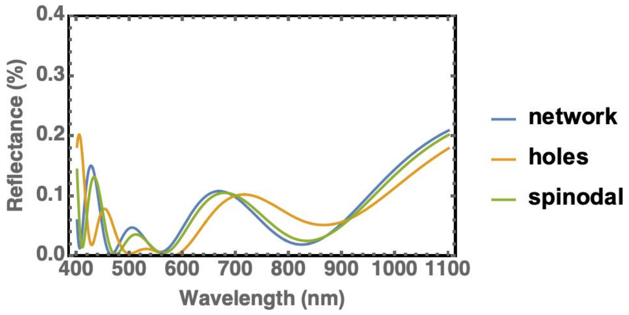

Fig. S4 Calculated reflectance for a representative double layer anti-reflection on c-Si, with the layers being the ideal ARC and the infiltrated pattern.

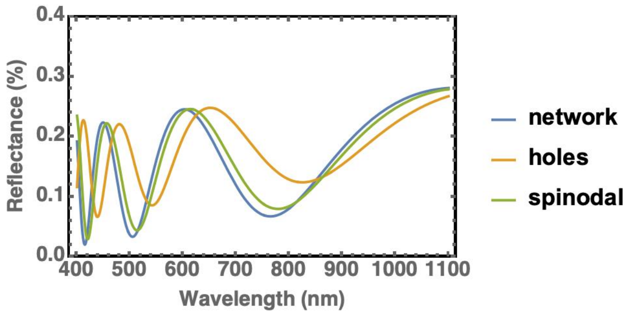

Fig. S5 Calculated reflectance for a representative double layer anti-reflection on c-Si, with the layers being the resist-infiltrated pattern and top resist layer of 50 nm

In our experiments, we have used a polymer resist as the low refractive index material, which has a sub-optimal refractive index ($n = 1.52$) and thickness ($h_{AR} \sim 50 - 100$ nm). The resulting reflectance (Fig. S5) is on average 10% higher compared to the previous case (Fig. S4).

Absorption spectra in the full device

The simulated Si absorption for the optimised HUD-based textures in a full device configuration (i.e. including the Ag back reflector and optimised design parameters listed in Table S1) are shown in the figure below.

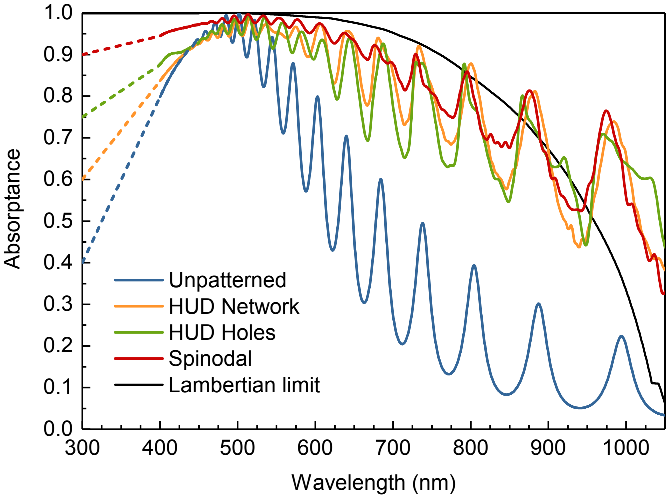

Fig. S6 *Simulated Si absorption spectra for the optimised textures in a full device configuration. The dashed lines indicate the range for which data was extrapolated.*

Si and Ag dispersion models

The dispersive dielectric function of c-Si was modeled using using a sum of Lorentzian terms[2]

$$\epsilon(\omega) = \epsilon_0 + \sum_{i=1}^{2} \frac{\sigma_i \omega_i^2}{\omega_i^2 - \omega_0^2 - i\omega\gamma_i} \,. \tag{1}$$

In principle, employing a large number of Lorentzian terms and/or splitting the spectral range[3] for the interpolation can provide an accurate fit for measured of the dielectric constant of silicon.[4] However, the running time for computing the absorption using the simplified Si absorption model for a $16 \times 16 \times 5\mu\text{m}^3$ cell using 300 computing cores and 1.2 Tb of memory, is about 12 hours. Increasing the number of Lorentzian terms and using multiple interpolation spectral ranges dramatically increase the computation time and make the

optimization process unpractical. To facilitate the optimisation process, we opt here for a simplified approach and use only two Lorentzian terms in the expansion with the values for the constants involved shown in Table S3.

Table S3 Parameters used to model the c-Si dispersion in Eq. 1.

	ε_0	ω_1	σ_1	γ_1	ω_2	σ_2	γ_2
c-Si	3.815	3.077	466.50	0.657	3.078	-458.431	0.669

While our model dielectric constant display a somewhat larger absorption coefficient in the long wavelength regime, it should provide a reliable ranking of the different designs and a reliable starting point for the fabricated structures. Comparing the Lambertian limited integrated AM1.5G absorption (IA) from for the "real" and "simplified" Si extinction coefficient, we find that our model overestimates the IA by 4% (absolute), which is equivalent to 1.6 mA/cm^2.

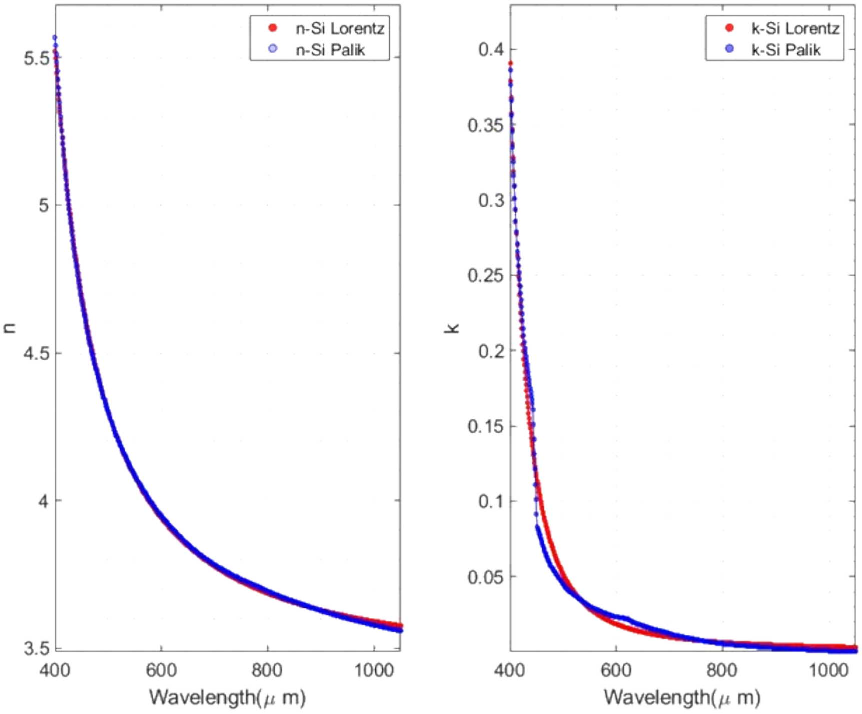

Fig. S7 Comparison of the real and imaginary part of the c-Si index of refraction used in the simulations (Lorentz label) against the values in Ref.[4] (Palik label).

The dielectric function of Ag was modeled using using the parameters from Ref.[2]

Table S4 Parameters used to model the Ag dispersion in Eq. 1.

	ε_0	ω_1	σ_1	γ_1
Ag	3.8451	$9.986 \cdot 10^{-21}$	$5.4821 \cdot 10^{41}$	0.0129

Coupled Mode Analysis

Light-trapping in slabs with thicknesses comparable to the wavelength of the incident light in general is accomplished by coupling to guided optical modes supported by the absorbing layer, which in case of weak absorption have propagation distances typically much longer than the thickness of the slab. The coupling to guided modes is achieved through roughening or corrugating the surface of the absorbing layer, which renders certain portions of the guided modes excitable by incident light, giving rise to guided resonances.[5] For the case of periodic patterns added to the slab's surface, it was shown that the $4n^2/\sin\theta$-limit calculated for a Lambertian scatterer can be surpassed, when the grated structure exhibits sub-wavelength modal confinement.[6] The analysis is based on temporal coupled-mode theory[7] and applied to the case of nanophotonic light-trapping. The ultimate limit of achievable absorption corresponds to the normalised sum over the absorption spectra of all guided resonances present in the frequency interval of the incident light.

We start with the equation governing for the amplitude of a given single guided resonance:[6]

$$\frac{d}{dt}a(t) = \left(i\omega_0 - \frac{N\gamma_e + \gamma_i}{2} \right) a + i\sqrt{\gamma_e}S(t) \ , \tag{2}$$

with a the resonance amplitude (normalised such that, $|a|^2$ is the energy per unit area in the slab), ω_0 the resonance frequency, N the number of excitation channels that a given resonance can couple to, γ_e the extrinsic loss-rate to each of the N channels, γ_i the intrinsic loss

rate of the resonance due to material absorption, and S the amplitude of a given excitation channel. Assuming a harmonic expansion for the resonance and the incident wave amplitudes, $a(t) = a(\omega)\exp(i\omega t)$, $S(t) = S(\omega)\exp(i\omega t)$, the absorption spectrum of the resonance is given by

$$A(\omega) \equiv \frac{\gamma_i(\omega)\,|a(\omega)|}{|S(\omega)|} = \frac{\gamma_i\gamma_e}{[\omega-\omega_0]^2 + [\gamma_i + N\gamma_e]^2/4}, \tag{3}$$

The spectral cross-section of the resonance is found through the integral $\sigma = \int_{-\infty}^{\infty} d\omega A(\omega)$, which, under the assumption of weak variation of N, γ_e, and γ_i with frequency, is given by

$$\sigma = 2\pi\gamma_i \frac{1}{N + \gamma_i/\gamma_e}. \tag{4}$$

In the overcoupling regime $\gamma_e \gg \gamma_i$, or in case $N \gg \gamma_i/\gamma_e$, the maximal value of Eq. (4) can be approximated as:[6]

$$\sigma_{\max} = 2\pi\gamma_i/N \tag{5}$$

This maximum spectral cross-section carries the unit of frequency and when normalized by the incident spectral bandwidth $\Delta\omega$, it is assumed that the resonance contributes an additional $\sigma/\Delta\omega$ to the absorption $\bar{A}$. The value of $\bar{A}$ is then found as the sum over the maximum spectral cross-section of all modes, normalised by the incident spectral bandwidth $\Delta\omega$:

$$\bar{A} = \frac{\sum \sigma_{\max}}{\Delta\omega} = \frac{1}{\Delta\omega}\sum_m \frac{2\pi\gamma_{i,m}}{N_m}\ , \tag{6}$$

where the index m labels individual resonances and the summation is performed over all modes in the frequency interval $\Delta\omega$. When the guided resonance is approximated as a plane wave in the slab, the intrinsic loss-rate can be expressed as $\gamma_{i,m} = \alpha_m \cdot v_m$, where α_m is the mode's absorption coefficient $\alpha_m = 2\frac{\omega}{c}\kappa_m$. Here, κ_m and v_m and denote the imaginary part of the mode's refractive index and its group velocity, respectively.

We now apply this formalism to the physics of our HUD membranes. Here the modes

m are the guided modes supported by the slab (dispersing as in Fig. 2a of the main text), while the channels N_m that they are subject to is determined by the structure factor of the surface texture.

The number of channels N associated with a given mode are obtained by considering the structure factor of the surface texture. We approximate the structure factor as a ring in reciprocal space of homogeneous intensity with inner radius k_1 and outer radius k_2 and assume that waves scattered by the surface patterning acquire any wavevector exclusively within this range with equal probability. From the k-space area of the structure factor $\mathcal{A} = \pi\left(k_2^2 - k_1^2\right)$ relative to the area of a single mode $\mathcal{A}_k = 2\pi k_m \Delta k_m$, the number of channels can then be approximated as

$$N_m = \frac{\mathcal{A}}{\mathcal{A}_k} = \frac{\left(k_2^2 - k_1^2\right)}{2k_m \Delta k_m}, \tag{7}$$

with the mode's propagation constant $k_m = \dfrac{\omega}{c} n_m$, the mode's refractive index n_m and where the spectral width of the resonance Δk_m depends on the structure factor as well as the incident bandwidth $\Delta\omega = c\Delta k$, with speed of light c. The case of a continuous structure factor as considered here, in contrast to the discrete points in reciprocal space corresponding to a periodic grating, bears the consequence that the absorption spectrum in the vicinity of a resonance is not necessarily in the form of a sharp Lorentzian, but instead can have significant absorption over a larger wavelength interval. From the broad shape of the resonance in the HUD case shown in Fig. S8, we infer that a single average value will not provide a faithful representation of the absorption and integration over the spectral width Δk_m will result in a more sensible approximation. For this case, the summation over the guided resonances is performed for each small wavelength interval and the result normalised by the spectral bandwidth of the resonance $\Delta\omega_m = c\Delta k_m$. Furthermore, the mode's group velocity $v_{\mathrm{g}} = \dfrac{c}{n + \omega \dfrac{\partial n}{\partial \omega}}$ approaches the phase velocity $v_{\mathrm{p}} = \dfrac{c}{n_m}$, since the mode index variation vanishes in the small wavelength interval limit, such that the internal loss-rate can be approximated as $\gamma_{i,m}(\omega) = \alpha_m(\omega) \cdot v_{\mathrm{g}}(\omega) \approx \alpha_m(\omega)\,\dfrac{c}{n_m}$. Substituting N and γ_i in Eq. (6) and

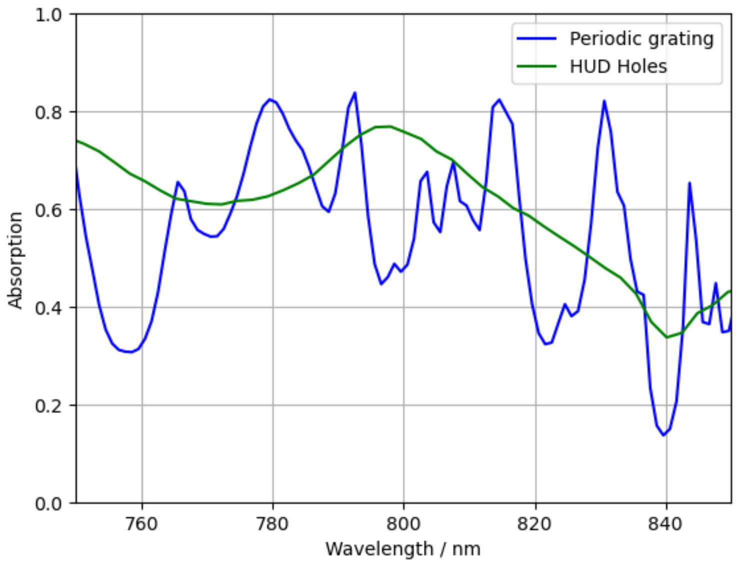

Fig. S8 Excerpt of the absorption spectrum for an optimized periodic grating compared with a stealthy hyperuniform point distribution, obtained from FDTD simulations.

rewriting the same for the narrow band limit $\Delta\omega \to c\Delta k_m$, we obtain:

$$\tilde{A}_{\mathrm{T}}^{\omega} = \sum_m \frac{4\pi\alpha_m k_m}{n_m(k_2^2 - k_1^2)} \ , \tag{8}$$

where superscript ω indicates that α_m, k_m, and n_m are now evaluated at the frequency ω under consideration and not averaged across the resonance's spectral width. Equation 8 clearly highlights the influence of the structure factor on the absorption in the slab. The $k_{1,2}$-ring area relates to the number of modes that couple to a single resonance and reducing it on the one hand increases the overall absorption. On the other hand, the number of modes taking part in the summation is also reduced, leading to a lower overall absorption. This has an important impact in the broadband regime where there is a large ensemble of relevant modes for absorption as shown in Fig. 2 in the main manuscript. Conversely, in the narrow band regime, where the number of relevant modes is comparatively small, it is possible to reduce the area of the structure factor without greatly disturbing the number of modes in the summation.

Furthermore, we conjecture that the $\tilde{A}_{\mathrm{T}}^{\omega}$ is representative of the frequency-dependent

absorbance of the film (defined as $\tilde{\alpha}(\omega) \times \ell$, with $\tilde{\alpha}(\omega)$ the effective absorption coefficient and ℓ the sample thickness). We consider that this phenomenological approach provides a good starting point in our optimisation process and we emphasise that all designs derived here are further optimised and validated with full-wave 3D FDTD simulations.

In order to calculate an upper limit for the total absorption of light in the slab, we employ the analytical solutions derived for Lambertian light-trapping in textured solar cells,[8] which considers a thin absorber with the texture on the front-, and a perfect metal reflector on the rear surface. The absorption expression is reproduced for convenience below:

$$A_{\mathrm{T}}(\omega) = \frac{1 - \exp\left(-f_p \cdot \tilde{A}_{\mathrm{T}}^{\omega}\right)}{1 - (1 - \frac{1}{n}) \exp\left(-f_p \cdot \tilde{A}_{\mathrm{T}}^{\omega}\right)}, \tag{9}$$

where the product absorption coefficient times slab thickness $\alpha\ell$ is identified with Eq. (8) as indicated above. The calculated path-length enhancement factor $f_p = 4$ in the weakly absorbing limit results in an over-estimation of the absorption in the small-wavelength range, but is retained nonetheless to facilitate computation of the spectrally averaged absorption. The latter is found through convolution of Eq. (9) with the AM1.5 photon flux and integration over the range $[400, 1050]$ nm, normalized to the total amount of photons in the same interval:

$$IA = \left[\int_{\omega} F(\omega)\mathrm{d}\omega\right]^{-1} \cdot \int_{\omega} A_T(\omega)F(\omega)\mathrm{d}\omega, \tag{10}$$

where $F(\omega)$ describes the number of photons per square meter and second at the frequency ω as obtained from the AM1.5 spectrum.

Figure S9 shows the spectrally averaged absorption as a function of k_1 and k_2, approximated by Eq. 10; we obtain that the absorption is maximised for $k_1 = 9.1$ µm^{-1} and $k_2 = 24.8$ µm^{-1}, but maintains relatively large values for a range of k values around the optimal values.

The optimal values for k_1 and k_2 can be further refined by taking into account the

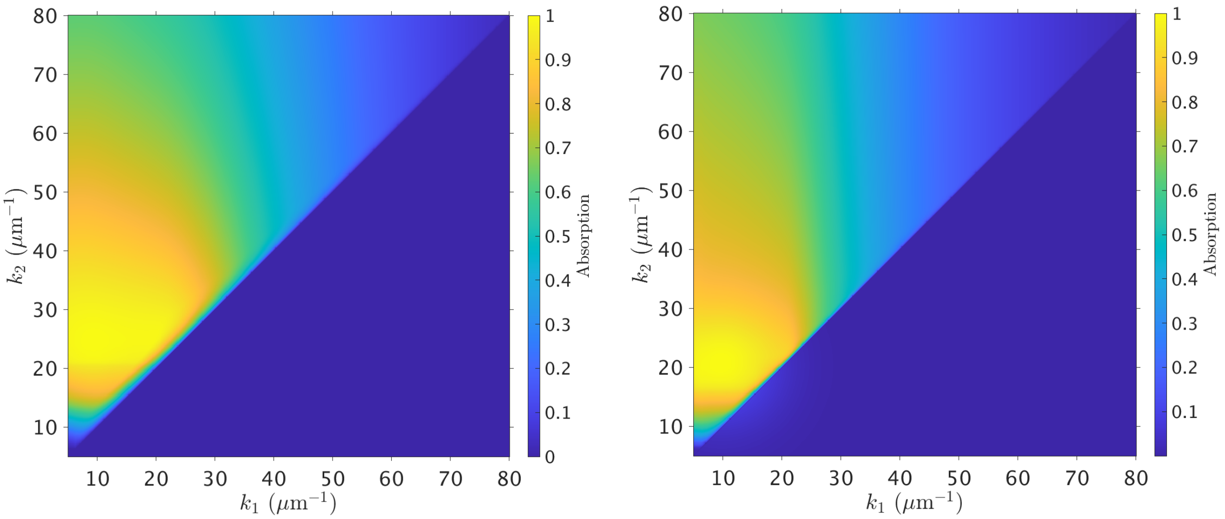

Fig. S9 Absorption of a Si slab as a function of the inner (k_1) and the outer (k_2) radius of the ring-shaped homogeneously distributed structure factor, using the spectrally averaged absorption over the frequency range 400 − 1050 nm. Left: Mode data obtained through a finite-difference eigenmode solver for a slab with metal back-reflector in air. Right: Similarly obtained mode data for a slab with back-reflector, anti-reflective coating ($h_{AR} = 72$ nm, $n_{AR} = 1.82$) and including Maxwell-Garnett effective medium approximation for the patterned layer.

anti-reflecting coating ($h_{\mathrm{AR}} = 72$ nm, $n_{\mathrm{AR}} = 1.82$) and the corrugated surface by using an effective homogeneous medium with a complex refractive index calculated using Maxwell-Garnett effective medium theory for a slab of thickness 196 nm. Under these assumptions, the intensity is now maximised for $k_1 = 9.7$ μm^{-1} and $k_2 = 20.4$ μm^{-1}.

For the $k_{1,2}$-values identified above, the effective absorption coefficient absorption, $\tilde{\alpha}0(\omega) \times \ell$ is compared with the bulk absorption coefficient times thickness, $\alpha_0(\omega) \times \ell$, to identify the predicted absorption enhancement across the spectral bandwidth.

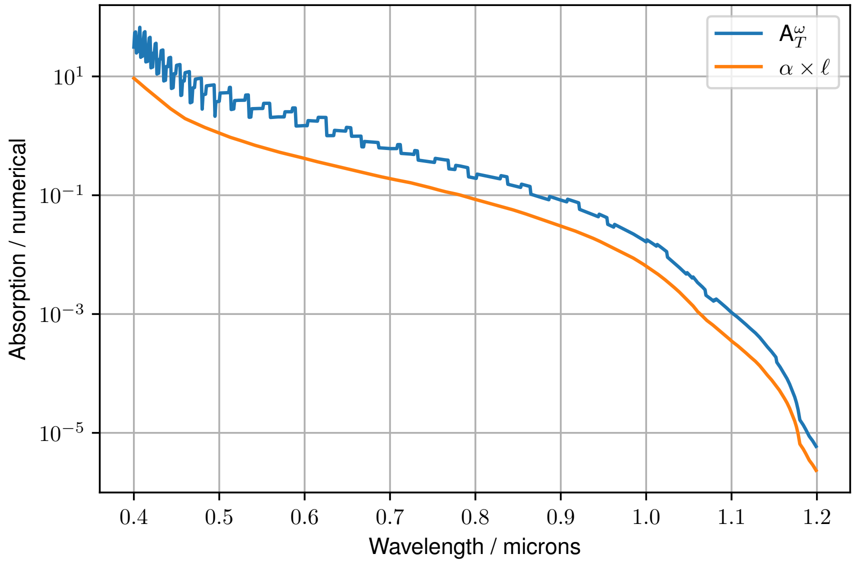

Fig. S10 Comparison of Eq. (8) with $\alpha_0 \ell$, the product of bulk absorption coefficient with slab thickness ℓ.

From the trends in Fig. S10 enhanced throughout the entire spectral bandwidth, especially so in the low-wavelength regime, while the absorption approximates the product $\alpha_0(\omega) \times \ell$ close to the bandgap of silicon. The fact that broadly speaking the blue curve traces the orange curve up to a multiplicative factor (vertical additive offset on log scale in Fig. S10) emphasizes that the effect of the HUD can be viewed as an effective absorption coefficient enhancement.

Angular dependence of absorption

In this section we contrast the angular dependence of the absorption in the samples analysed. The results presented in Fig. S11b show that the periodic structuring gives rise to a strongly anisotropic absorption, whereas the for a disordered structuring (Fig. S12), be it hyperuniform or spinodal the angular response is mostly isotropic roughly following the unstructured slab (Fig. S11a) case but with an enhanced absorption due to the optimised coupling of the incoming solar radiation to the quasiguided modes of the silicon slab.

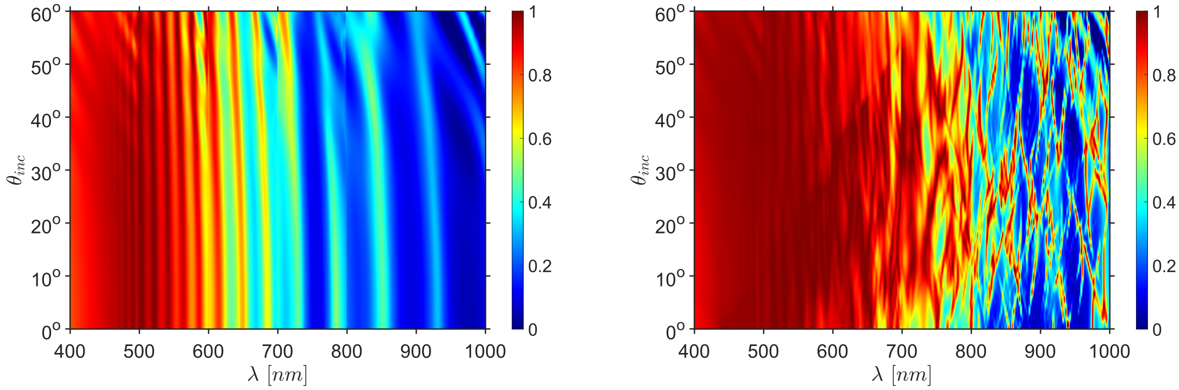

Fig. S11 Angular dependence of the absorption for (left) an unstructured 1 μm-thick silicon slab and (right) the periodically structured texture in a 1μm-thick silicon slab.

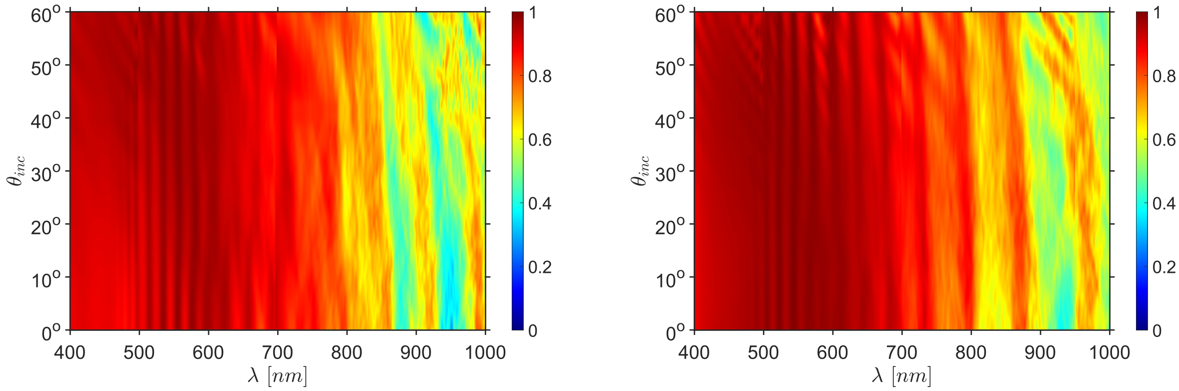

Fig. S12 Angular dependence of the absorption for (left) a hyperuniform structured texture and (right) the spinodal structured texture in a 1μm-thick silicon, respectively.

Power Spectral Density robustness to fabrication imperfections

We have checked the effect of the fabrication conditions on the power spectral density (PSD) of the scattering patterns through Fourier transforming SEM images of the samples. We have fabricated 100x100 μm^2 areas of the three designs under different electron doses in the exposure step of e-beam lithography (indicated by the dose factor DF, where DF=1 corresponds to a dose of 100 $\mu C/cm^2$). Similar to the experimental results, the HUD network is a very robust design, where the different fabrication parameters leads to a very similar PSD for k<20 μm^{-1} (i.e. the accessible range in our experiments). By contrast, the PSD

for the spinodal design is in particular highly sensitive to fabrication conditions, especially at k<10 µm^{-1}. In the case of the spinodal design, increasing the dose reduces the filling fraction of Si in the nanotexture and induces the increase of scattering strength below the desired diffraction ring in the wavevectors $k < 20\mu m^{-1}$. We have noted that this is only true in the spinodal design and we believe that it is intrinsic to the nature of the spinodal pattern. Changing the Si filling fraction essentially modifies the cutting threshold ϕ_0 that was used to optimise the pattern.

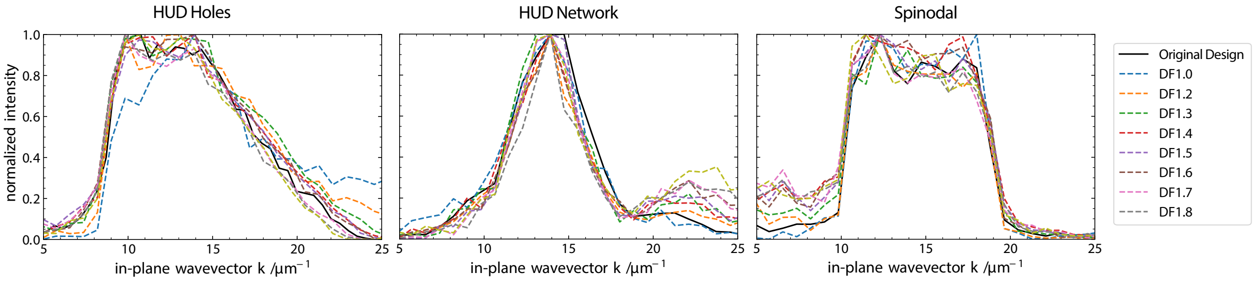

Fig. S13 Azimuthally averaged power spectral density (PSD) of the original design (black solid curve) compared to those of samples fabricated under different electron beam dose exposures (dashed curves), indicated by the dose factor, DF $\cdot 100\,\mu C/cm^2$. The experimental PSD is found by taking the square of radially-averaged magnitude maps of Fourier transformed SEM images.

Device Simulation Parameters in PC1D

To estimate the 1 µm silicon solar cell device performance, we have used the same 1D device model as in the Advanced HE-Tech devices recently reported by Buonassisi's group.[9] Despite the fact that in practice IBC cells require more complex 2D-3D architectures, this 1D model replicates well the performance of existing cells: 26.1% p-type IBC solar cell and 26.3–26.7% n-type IBC solar cells with Si heterojunction HIT architecture. We have also validated our 1D model system by comparing it to 2D simulations for thin IBC cells found in literature. We find that by using the same parameters in our 1D scheme and modified FSF and BSF doping profiles to thin Erfc functions (5 nm depth factor), we obtain virtually the same PV performance values (See Table S5).

Table S5 Comparison between the simulated parameters for two IBC solar cells with a 2D model and our 1D approach. The 2D simulation performance values are taken from Ref. 10.

3 µm thick cell	Sci. Rep. 2D model	Our 1D model
Jsc (mA/cm2)	39.3	39.3
Voc (V)	0.815	0.810
Efficiency (%)	28.2	27.8

10 µm thick cell	Sci. Rep. 2D model	Our 1D model
Jsc (mA/cm2)	43.6	43.6
Voc (V)	0.800	0.799
Efficiency (%)	30.8	30.6

In all simulations, we have considered Auger recombination with parameters from Richter et al.,[11] using PC1Dv6.1. All parameters to calculate the values in Figure 4 of the main text, are displayed in Table S6. We note however that the effect of Auger recombination is found to be negligible (about 0.01% efficiency decrese) here given the relatively low bulk lifetime. Therefore, Auger recombination was neglected in the analysis in the following section.

In here, the contact SRV is indicated by the back surface recombination parameter, while the front surface recombination parameter is effectively accounting for recombination at the passivated front surface (modulated by the emitter peak doping and front SRV). The low SRV at the contact is possible through recently reported advances in passivation schemes at the Si-metal contact for high efficiency PV (see for instance Ref. 12). For the front surface, we have used larger SRV values compared to best passivation schemes in flat Si, to account for the complexity of passivating vertical walls in Si. We also extensively discuss the effect of all these parameters in the following section. Moreover, for the three different structures (HUD network, HUD holes and spinodal) the front SRV parameter was multiplied by an SRV factor to account for the additional surface area generated through texturing.

Light trapping by our textures was introduced by tuning the internal reflection parameter in the PC1D model so that J_{photo} matches that from the integration absorption spectra (from 300 to 1050 nm) in the ideal device case (i.e. with minimal recombination losses). The front external and rear internal (specular) reflectances were fixed to 1.5% and 99.45%,

respectively, to account for the average front reflectance and metal (specular) back-relfection in our devices. The internal (diffuse) reflectance was modified equally for first and subsequent bounces. The used internal reflection and a SRV enhancement factors are presented in Table S7, along with the obtained short circuit current (J_{sc}) open-circuit potential (V_{oc}) and efficiency (η) for all the different patterning designs. The corresponding I-V and efficiency curves for all four surface texturing are displayed in Figure S14.

Table S6 Parameters used for the PC1Dv6.1 simulations of the 1 μm Silicon solar cell.

Constant parameters	Value
Exterior rear reflectance	0%
Base resistance	20 Ωcm^2
Emitter (peak doping and Erfc depth factor)	6 x 10^{18} cm^{-3}, 5 nm
Background doping	7x10^{15} cm^{-3}
Bulk recombination	500 μs
Rear surface recombination	1 cm/s
Front surface recombination	100 cm/s
Auger recombination model	Richter et al. 2012
Bandgap renormalisation	Yan&Cuevas, 2013
Mobility	Schindler et al. 2014

Table S7 Results of PC1Dv6.1 simulations with the values presented in Table S6. Current densities are in units of (mA/cm^2)

	SRV factor	Internal reflection	V_{oc} (V)	J_{sc}	Efficiency (%)
Unpatterned	1	25	0.768	18.4	11.9
HUD holes	1.75	94	0.770	32.4	21.2
HUD Netw.	3.274	94.3	0.755	32.6	20.9
Spinodal	2.37	96	0.764	33.8	22.0

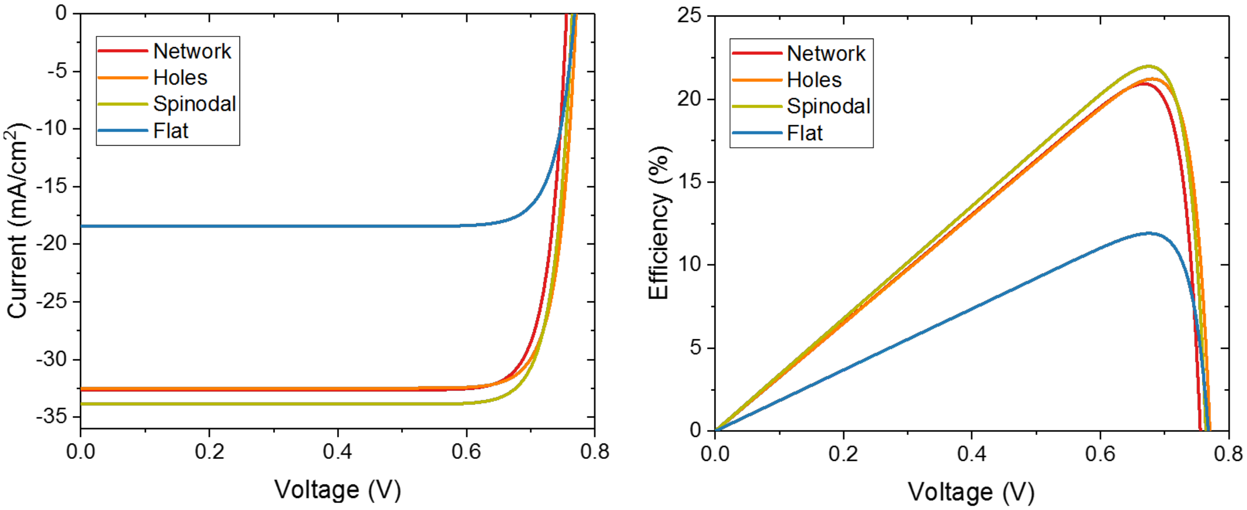

Fig. S14 *I-V curves (a) and efficiency curves (b) corresponding to different front textured surfaces. The colors represent the different structures.*

Bulk lifetime and surface recombination effects

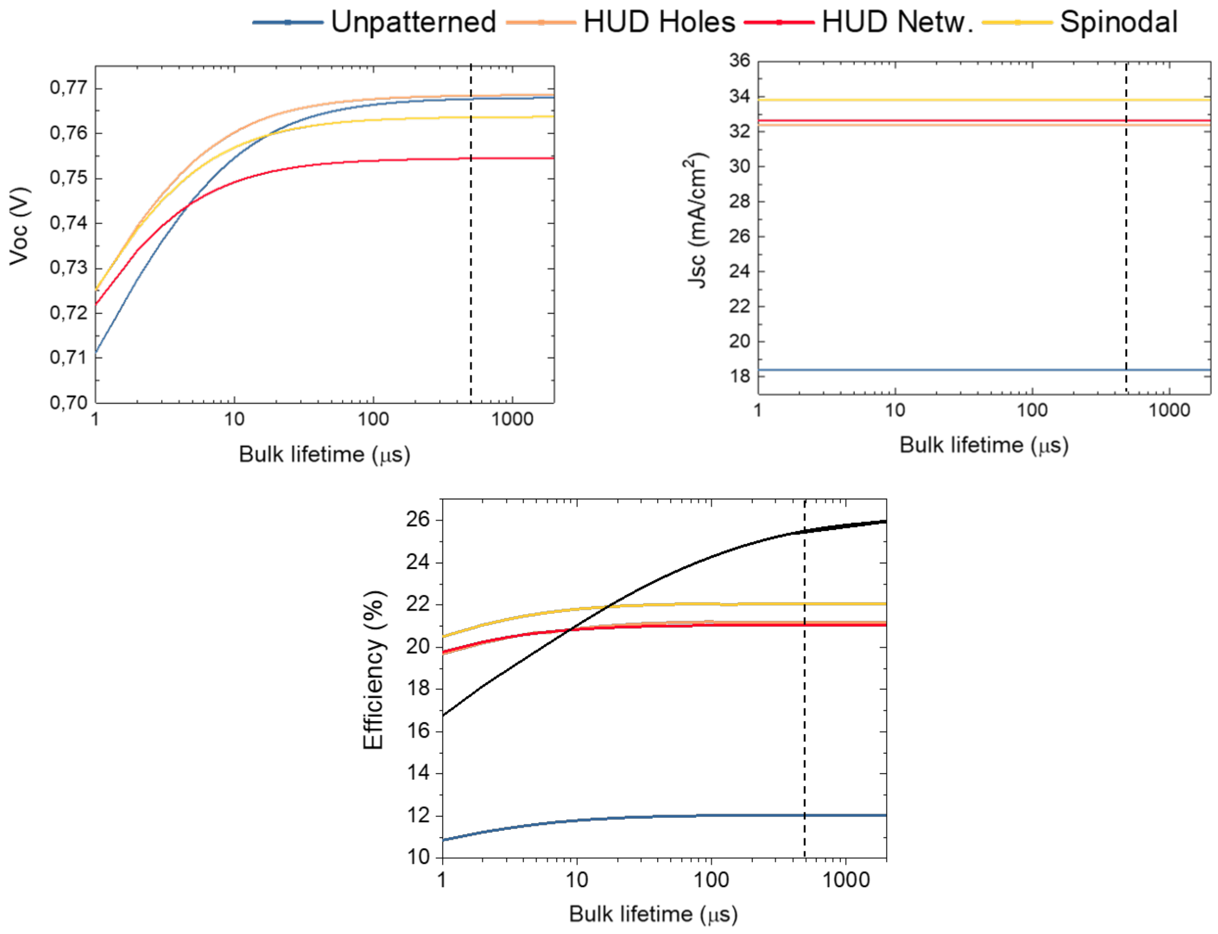

Fig. S15 Efficiency plotted against the logarithm of bulk recombination lifetime for the four different structures. The efficiency plot also contains efficiency of bulk 200 μm silicon as reference (black line).

A comparison of the 1 μm Si membrane with the presented standard values to other devices is made by adjusting several parameters in PC1D. Firstly, efficiencies of the four devices are calculated with a range in bulk recombination lifetime (τ) of 100 μs to 2 ms, plotted in Figure S15. Different bulk lifetimes translate to the quality of silicon as high quality silicon has a long bulk lifetime and vice versa. In order to have the 1 μm device into perspective with a 200 μm solar cell, PC1D was again used with the same parameters as in Table S6.

Figure S15 clearly shows that the efficiency in any of the thin film cells is hardly affected by increasing the bulk recombination lifetime from 100 μs to 2000 μs. This observation is

explained by the fact that a 1 µm silicon membrane is too thin for bulk recombination to play a major role in recombination losses. Bulk lifetime thus has little to no influence on thin film Si and bad quality silicon can therefore be used for thin films without a major loss in efficiency.

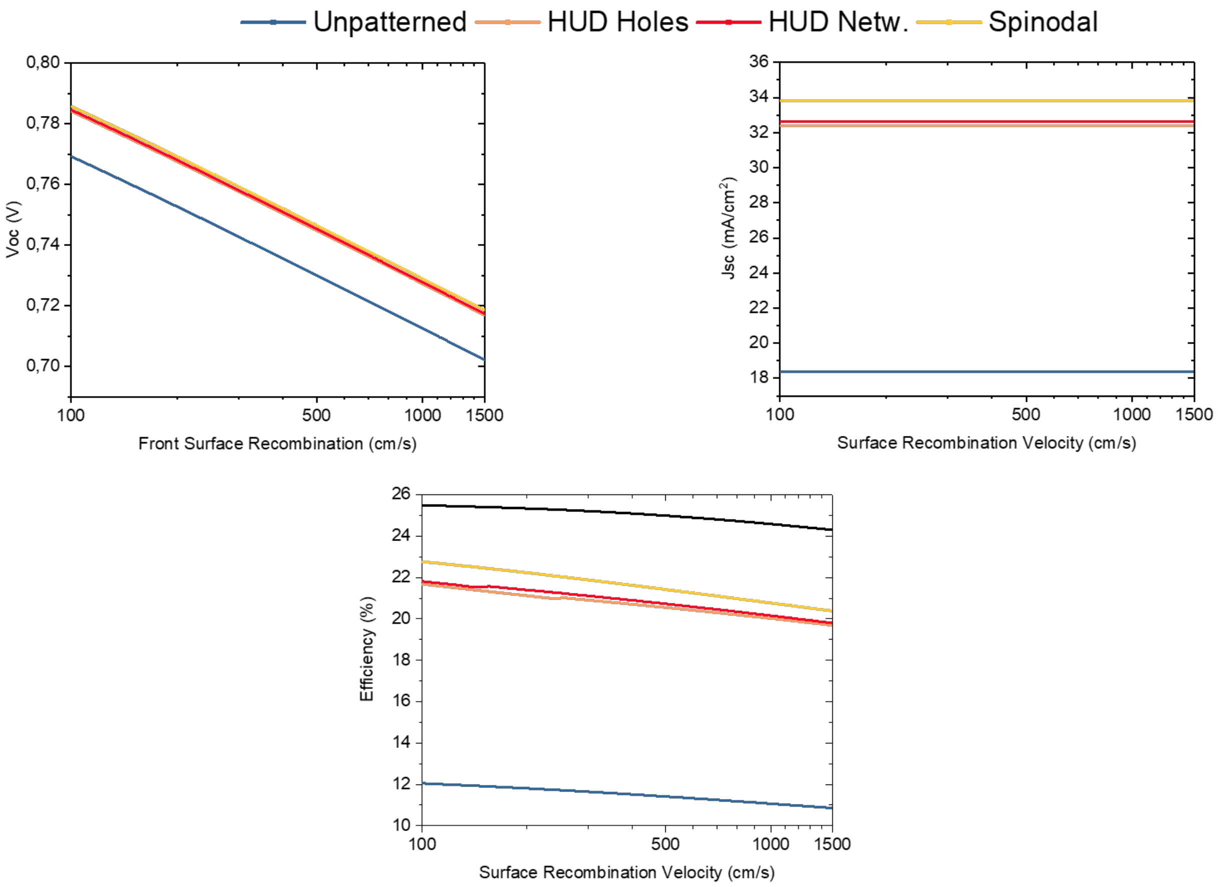

Fig. S16 Efficiency plotted against the logarithm of SRV values for the four different structures. The efficiency plot also contains efficiency of bulk 200 µm silicon as reference (black line).

On the other hand, an increase in lifetime in a 200 µm Si wafer does affect efficiency significantly, particularly when the bulk lifetime becomes smaller than 500 µs. The efficiency drops by 1.7% absolute efficiency by increasing the bulk lifetime from 100 to 2000 µs. For thicker wafers it is therefore necessary to use silicon with the highest possible quality to minimize bulk recombination losses.

Figure S16 shows how is the efficiency affected by the surface recombination velocity

(SRV) at the front surface. We observe that increasing the SRV induces an efficiency drop in all devices, patterned, unpatterned and bulk. An increase in SRV from 100 to 1000 for the flat surface results in a decrease in efficiency of about 1% absolute. With texturing and therefore extra surface, the decrease in efficiency for the patterned thin films is about twice as much. Again, this can be explained by the fact that the surface area to volume ratio for a 1 μm silicon membrane is very large and the surface recombination dominates.

Table S8 summarizes the solar cell performance in a bulk Si cell for two extreme cases of bulk lifetime (0.5 and 5 ms) and SRV (100 and 1000 cm/s). For the rest of the electronic parameters, we have used the values listed in Table S6. For the sake of comparison, we have also considered the same light trapping and enhanced surface area given by the different nanopattern designs. Because of the small effect of SRV to the total efficiency in bulk Si, the V_{OC} is the same (within two decimal spaces) for all the designs.

Table S8 Results of PC1D simulations for a 200 μm Silicon solar cell. For comparison an SRV of 100 cm/s and bulk lifetime of 500 and 5000 μs (top), and bulk lifetime of 500 μs and SRV of 100 and 1000 cm/s (bottom) are displayed in the same table.

τ=500 μs

	SRV=100 cm/s			SRV=1000 cm/s		
	V_{oc} (V)	J_{sc} (mA/cm^2)	η (%)	V_{oc} (V)	J_{sc} (mA/cm^2)	η (%)
Unpatterned	0.744	40.2	25.5	0.722	40.2	24.5
HUD Holes	0.743	43.01	27.3	0.714	43.0	26.0
HUD Netw.	0.739	43.1	27.1	0.700	43.1	25.5
Spinodal	0.744	43.3	27.4	0.708	43.3	25.9

SRV=100 cm/s

	τ=500 μs			τ=5000 μs		
	V_{oc} (V)	J_{sc} (mA/cm^2)	η (%)	V_{oc} (V)	J_{sc} (mA/cm^2)	η (%)
Unpatterned	0.744	40.2	25.5	0.753	40.3	26.1
HUD Holes	0.743	43.01	27.3	0.751	43.1	27.8
HUD Netw.	0.739	43.1	27.1	0.745	43.4	27.9
Spinodal	0.744	43.3	27.4	0.745	43.2	27.6

Figure S17 shows V_{oc} and efficiency as a function of thickness. V_{oc} first increases with

increasing thickness and after approximately 10 microns it decreases constantly due to more recombination mechanisms in bulk material. However, efficiency increases in the first part rapidly and around 30 microns flattens out to a relatively constant value. This increase in efficiency is mostly caused by an increased Jsc which in turn is the result of more light absorption in bulk material.

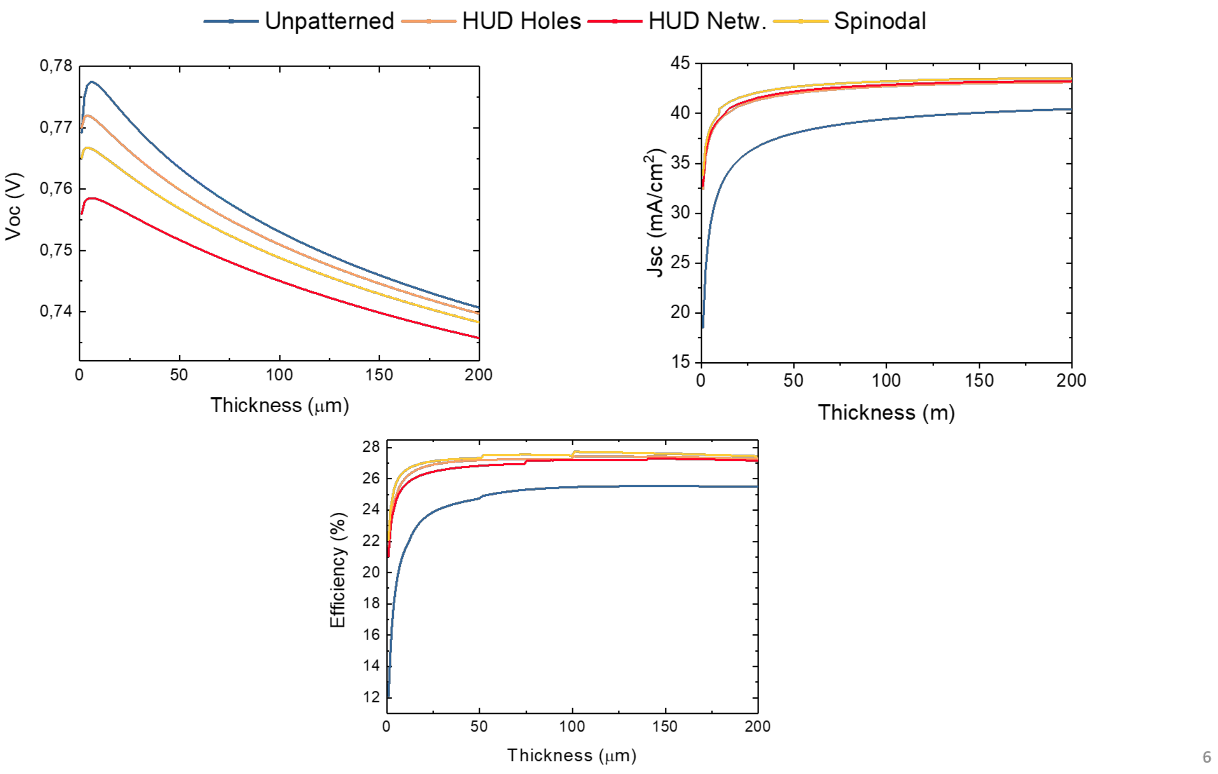

Fig. S17 *Open-circuit voltage V_{oc}, short-circuit current density J_{sc} and solar cell efficiency for p-type silicon with the four different surface texturing plotted against thickness.*

Integrating sphere set-up

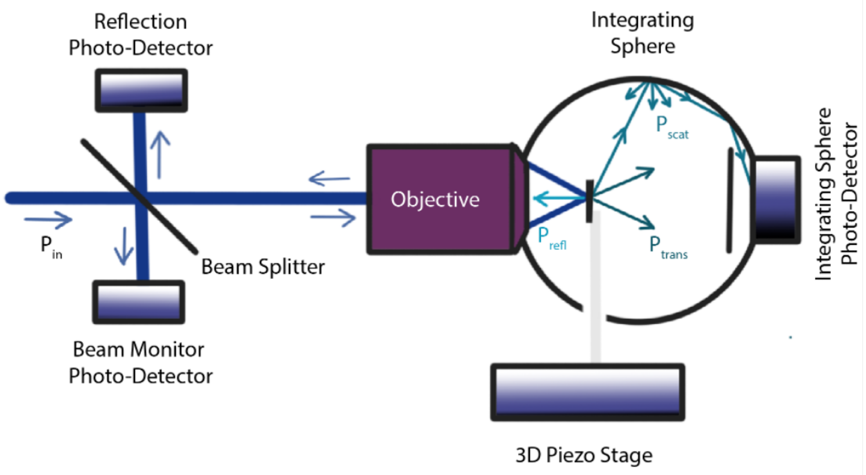

Fig. S18 *Sketch of the integrating sphere set-up for the absorption measurements.*

Figure S18 shows a sketch of the absorption measurement set-up as described in the Methods section and in Ref. 13. Absorption is determined from back-scattered, transmitted and specularly reflected light through carefully calibrated "Integrating Sphere Detector" and "Reflection Detector". The sample (black rectangle) is located in the center of the integrating sphere, and its position is adjusted with a piezo stage.

References

(1) Wang, E. Y.; Yu, F. T.; Simms, V. L.; Brandhorst, H. W.; Broder, J. D. Optimum design of antireflection coating for silicon solar cells. 1974; pp 168–173.

(2) Pratesi, F.; Burresi, M.; Riboli, F.; Vynck, K.; Wiersma, D. S. Disordered photonic structures for light harvesting in solar cells. Optics Express **2013**, 21, A460.

(3) Deinega, A.; John, S. Effective optical response of silicon to sunlight in the finite-difference time-domain method. Optics Letters **2011**, 37, 112.

(4) Palik, E. D., Ed. Handbook of optical constants of solids II; Academic Press: Boston, 1991.

(5) Fan, S.; Joannopoulos, J. D. Analysis of guided resonances in photonic crystal slabs. Physical Review B **2002**, 65.

(6) Yu, Z.; Raman, A.; Fan, S. Fundamental limit of nanophotonic light trapping in solar cells. PNAS **2010**, 107, 17491–17496.

(7) Haus, H. Waves and fields in optoelectronics; Prentice-Hall: Englewood Cliffs, NJ, 1984; pp 198–230.

(8) Green, M. A. Lambertian light trapping in textured solar cells and light-emitting diodes: analytical solutions. Prog. Photovolt: Res. Appl. **2002**, 10, 235–241.

(9) Liu, Z.; Sofia, S. E.; Laine, H. S.; Woodhouse, M.; Wieghold, S.; Peters, I. M.; Buonassisi, T. Revisiting thin silicon for photovoltaics: a technoeconomic perspective. Energy & Environmental Science **2020**, 13, 12–23.

(10) Bhattacharya, S.; John, S. Beyond 30% Conversion Efficiency in Silicon Solar Cells: A Numerical Demonstration. Sci. Rep. **2019**, 9, 12482.

(11) Richter, A.; Glunz, S. W.; Werner, F.; Schmidt, J.; Cuevas, A. Improved quantitative description of Auger recombination in crystalline silicon. Phys. Rev. B **2012**, 86, 165202.

(12) Collett, K. A.; Bonilla, R. S.; Hamer, P.; Bourret-Sicotte, G.; Lobo, R.; Kho, T.; Wilshaw, P. R. An enhanced alneal process to produce SRV $<$ 1cm/s in 1Ωcm n-type Si. Sol. Energy Mater. Sol. Cells **2017**, 173, 50–58.

(13) Mann, S. A.; Sciacca, B.; Zhang, Y.; Wang, J.; Kontoleta, E.; Liu, H.; Garnett, E. C. Integrating Sphere Microscopy for Direct Absorption Measurements of Single Nanostructures. ACS Nano **2017**, 11, 1412–1418.
